# Persistent bistability of Nordic Seas overflow waters over 800,000 years

**DOI:** 10.1126/sciadv.aee7886

**Published:** 2026-11-04

**Authors:** Nathan Stevenard, Aline Govin, Catherine Kissel, Didier Swingedouw, Samuel Toucanne, Nathaëlle Bouttes

**Affiliations:** ^1^ Laboratoire des Sciences du Climat et de l’Environnement, LSCE, CEA-CNRS-UVSQ, Université Paris-Saclay, Gif-sur-Yvette, France.; ^2^ Institut des Géosciences de l’Environnement, IGE, Université Grenoble Alpes, Saint-Martin d’Hères, France.; ^3^ Earth and Life Institute, Université catholique de Louvain, Louvain-la-Neuve, Belgium.; ^4^ Université de Bordeaux EPOC, UMR 5805, Pessac, France.; ^5^ Geo-Ocean, Université de Brest, CNRS, Ifremer, UMR 6538, Plouzané, France.

## Abstract

The stability of the Atlantic Meridional Overturning Circulation (AMOC) is a major uncertainty in projections of future climate. However, direct evidence for AMOC tipping behavior on orbital timescales remains scarce. Here, we reconstruct the strength of Iceland-Scotland Overflow Water, a key lower branch of the deep Atlantic circulation, over the past 800,000 years by combining 12 current-speed records from North Atlantic sediment cores with a data-driven hybrid model. The resulting reconstruction reveals persistent alternation between weak (glacial) and strong (interglacial) circulation modes, pervasive millennial-scale variability within both regimes, and systematic asymmetry between weak-to-strong recoveries and strong-to-weak collapses. Threshold and phase-space analyses show that regime shifts occur within a narrow window of atmospheric carbon dioxide and sea level and often follow hysteresis-like loops. Although bistability persists throughout the last 800,000 years, its sensitivity and timing evolve through time. These findings provide long-term, high-resolution, data-driven constraints on the bistability of Nordic Seas overflow waters and its implications for AMOC resilience in a warming world.

## INTRODUCTION

The Atlantic Meridional Overturning Circulation (AMOC) is a key component of Earth’s climate system, accounting for approximately 30% of the combined ocean-atmosphere meridional heat transport at 30°N (*1*). In the North Atlantic, warm upper-ocean waters flow northward, contributing about 90% of the oceanic heat transport in the Atlantic at 26.5°N (*2*). In the Nordic Seas, open-ocean convection in the Greenland Sea, dense water formation along the Arctic shelves, and the transformation of Atlantic water (*3*, *4*) lead to the formation of dense deep-water masses that return southward into the Atlantic basin, overflowing above the Greenland-Iceland-Scotland ridge through two main passages (*1*, *5*–*7*), the Denmark Strait Overflow Water (DSOW) and the Iceland-Scotland Overflow Water (ISOW; fig. S1). The latter spreads along multiple pathways in the subpolar North Atlantic (*6*–*8*) and contributes roughly one-third (∼5.8 ± 1.4 Sv) of the present-day North Atlantic Deep Water (NADW) (*9*, *10*), making it a key indicator of the deep circulation’s strength. Its relative contribution to NADW during past glacial and interglacial periods however, remains poorly constrained. Deep water formation, including ISOW, has persisted over the last 400,000 years (*11*), including during full-glacial conditions (*12*) and millennial-scale events (*13*).

The millennial-scale stability of the AMOC has long been debated, from conceptual box models (*14*–*16*) to fully coupled climate models (*17*, *18*), which suggest that overturning can exhibit multiple stable equilibria and hysteresis under changes in freshwater forcing. In this bistable framework, “stable” refers to circulation states that act as attractors, resisting small perturbations on centennial-to-millennial timescales for a given set of boundary conditions. Abrupt transitions thus arise from threshold crossings rather than intrinsic instability, when slowly varying freshwater inputs push the system across a bifurcation (*14*–*16*, *18*, *19*). Large freshwater perturbations associated with iceberg discharges (e.g., “Heinrich events”) can trigger abrupt transitions from a vigorous to a weak overturning mode, whereas a subsequent reduction in freshwater input may eventually restore a strong state. Beyond such events, models also produce internally generated millennial-scale AMOC oscillations under intermediate CO_2_ and ice-volume conditions (*18*, *20*–*26*), where both strong and weak states coexist and the realized circulation mode depends on the system’s history. Zhang *et al.* (*22*) also highlighted the role of orbitally induced self-oscillation of the AMOC under intermediate atmospheric CO_2_ level and ice volume, implying a dual effect of orbital forcing on millennial-scale variability.

This “bistable window” in the CO_2_–ice-volume phase space may play a key role in glacial-interglacial transitions by amplifying millennial-scale climate feedbacks during deglaciations or full glacial inceptions (*27*, *28*). Although originally developed to explain millennial-scale variability, the underlying bifurcation structure is controlled by slowly varying boundary conditions such as CO_2_ and ice-sheet volume (*20*–*27*). As these parameters evolve over glacial-interglacial timescales, they determine which weak, strong, or oscillatory AMOC regime is dynamically reached, thereby linking millennial-scale overturning variability to orbital-scale climate changes (*29*). However, the location of these threshold windows and the AMOC response vary widely among models (*21*, *24*, *26*), hindering efforts to identify robust stability boundaries on orbital timescales. Critically, orbital-scale AMOC bistability has not yet been demonstrated using observational data, owing to the absence of continuous, high-resolution reconstructions of the deep AMOC strength spanning multiple climate cycles. Independent reconstructions of the deep Atlantic circulation strength, such as ^231^Pa/^230^Th records (limited to the last ∼140,000 years), highlight ongoing debates regarding the strength and structure of the AMOC during the last glacial period (*30*–*35*). Regarding ISOW specifically, existing paleoceanographic reconstructions have largely focused on recent periods (*36*–*38*), while long-term variability across several climate cycles remains poorly constrained (*39*). Moreover, proxy-based reconstructions of deep-current strength in the North Atlantic often diverge in detail within a single archive, despite broadly consistent long-term trends (*11*), complicating direct comparisons with external forcing and tests of threshold-based theories of the deep North Atlantic’s stability.

Here, we address this gap by extracting an ISOW strength index from six proxies measured in two sediment cores (12 records) located along the ISOW pathway, providing a high-resolution (100-year) stack covering the last 380,000 years. We then extend this reconstruction back to 800,000 years using a hybrid modeling framework that combines linear regression with machine learning, capturing both large-scale linear controls and nonlinear interactions. This approach yields a continuous, data-driven reconstruction of ISOW dynamics across eight glacial-interglacial cycles, which we use to identify threshold behavior, hysteresis, and evolving sensitivities of one major component of the deep North Atlantic circulation to external forcing.

## RESULTS

### ISOW_data_ stack over the last 380,000 years

The ISOW_data_ stack is constructed from two sediment cores located along the ISOW pathway (fig. S1) combining 12 records of current strength (see Materials and Methods). We apply a Monte Carlo principal component analysis to combine these records within their uncertainties, yielding a composite signal representative of ISOW variability (see Materials and Methods). The resulting stack reveals marked alternations in ISOW strength across the last four glacial-interglacial cycles (Fig. 1A). High ISOW strength values (strong regime) consistently characterize interglacials, while glacials are marked by lower values (weak regime). Transitions between these states are abrupt, with no evidence for persistent intermediate plateaus (Fig. 1E). In contrast, global mean sea level (GMSL) (*40*) and atmospheric CO_2_ concentrations (*41*, *42*) evolve gradually between glacial and interglacial plateaus (Fig. 1, C to E), indicating a nonlinear response of ISOW strength to slowly varying boundary conditions.

**Fig. 1.**
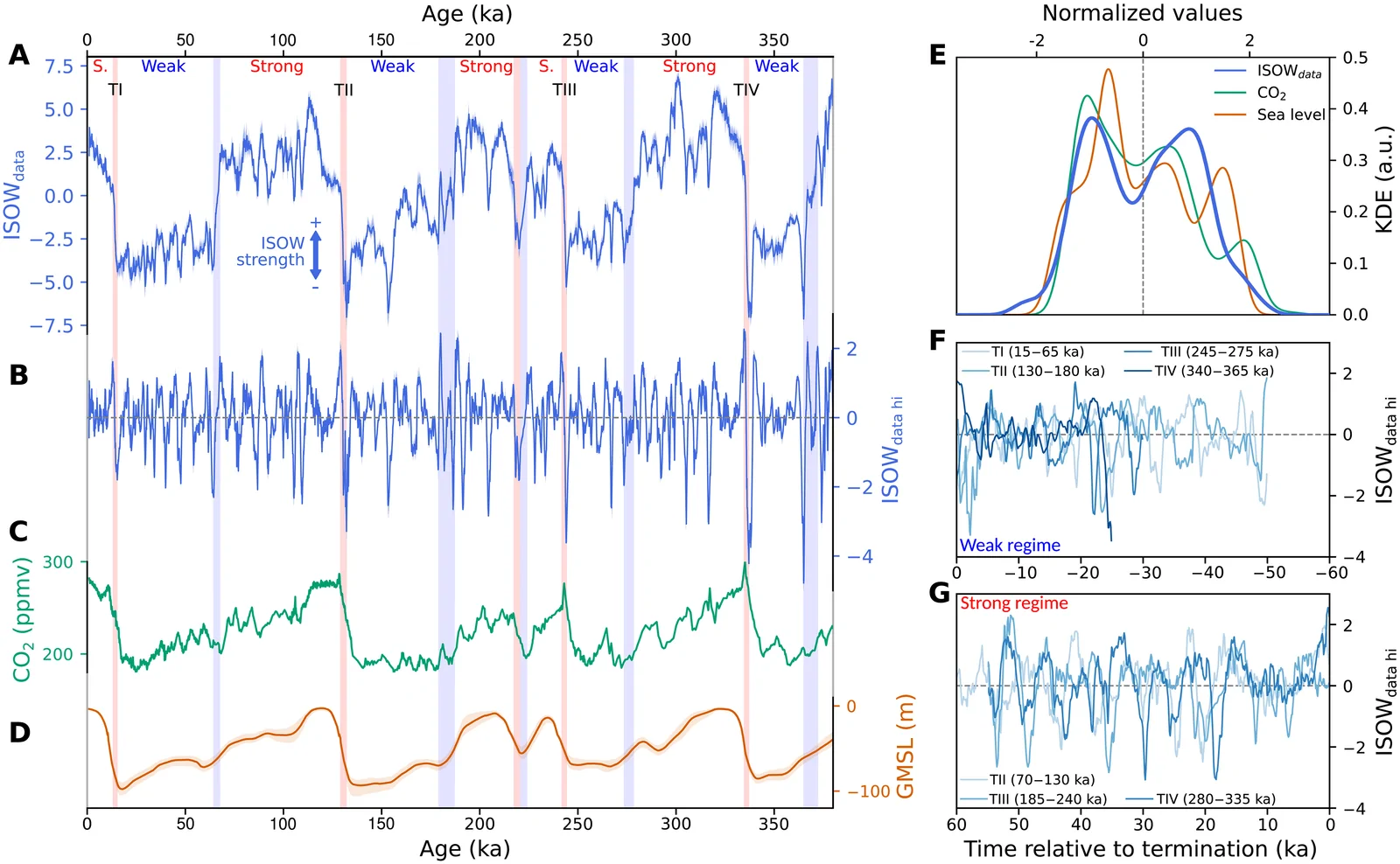
ISOW variability and global climate background over the last 380,000 years. (**A** to **D**) Time series of (A) ISOW strength (ISOW_data_), (B) millennial-scale variability (ISOW_data_ hi), (C) atmospheric CO_2_ concentrations (*41*, *42*), and (D) global mean sea level (GMSL) (*40*). ISOW_data_ hi is obtained following (*43*) by subtracting a 7-ka running mean from ISOW_data_, isolating the millennial-scale component. Vertical bars in (A) to (D) mark transitions between strong and weak ISOW regimes (red = weak to strong; blue = strong to weak), and numbers at the top denote termination numbers. (**E**) Kernel density estimates of *z*-score–normalized ISOW_data_, CO_2_, and GMSL values over the past 380,000 years. (**F** and **G**) High-pass ISOW anomalies (ISOW_data_ hi) within selected weak (F) and strong (G) ISOW regimes plotted as a function of time for a specific regime interval relative to each termination (TI to TIV). The time relative to terminations indicates the time before [i.e., weak regime; negative values in (F)] and after [i.e., strong regime; positive values in (G)] the transitions highlighted in (A) to (D) (light red vertical bars). Millennial-scale ISOW fluctuations of comparable amplitude and timing are present in both weak and strong regimes, indicating that vigorous overflow variability is a persistent feature of ISOW dynamics regardless of the regime.

Superimposed on this orbital-scale structure, the ISOW_data_ record also exhibits pronounced millennial-scale variability (Fig. 1B). After removing the orbital component of ISOW_data_ following the procedure described in (*43*), the residual signal displays millennial-scale fluctuations with comparable amplitude and timing during periods of weak and strong ISOW regimes, showing that millennial-scale variability is a persistent feature of the deep Atlantic circulation, largely independent of the circulation regime (Fig. 1, F and G).

### ISOW_hybrid_ stack over the last 800,000 years

To extend the ISOW_data_ stack beyond the last four climate cycles and test whether the bistable regime held under different climate backgrounds including cooler “lukewarm” interglacials that preceded the Mid-Brunhes Event (MBE) ∼430,000 years ago, we developed a hybrid model that combines a linear regression component trained on the ISOW_data_ stack with a machine learning component that captures nonlinear interactions among climate forcings (see Materials and Methods). Predictors (see Materials and Methods) include North Atlantic sea-surface temperature from planktic foraminifera counts and freshwater fluxes from Ice-Rafted Debris (IRD) counts (*44*), large-scale climate boundary conditions such as atmospheric CO_2_ concentrations (*41*) and benthic foraminifera δ^18^O as a first-order tracer for global ice volume (*45*), and orbital parameters (obliquity and precession) (*46*). Cross-validation and sensitivity experiments indicate that the model performs robustly across independent subsets of the data (tables S1 and S2 and fig. S2), with residuals centered around zero (see the Supplementary Materials; fig. S3). Sensitivity experiments (fig. S4) indicate that the full predictor set yields the most stable and parsimonious reconstruction. We further apply SHapley Additive exPlanations (SHAP) (*47*) (see Materials and Methods), a model-agnostic attribution method, to quantify the relative influence of each driver over the last 800,000 years.

The extended reconstruction indicates that ISOW remained organized into two distinct modes throughout the past 800,000 years: a strong state during interglacials and a weaker state during glacials (Fig. 2A). Transitions between these regimes are consistently sharp, with weak-to-strong recoveries occurring about 2.5 times faster (on the order of ∼2000 years, visually assessed from Fig. 2A) than subsequent strong-to-weak collapses (on the order of ∼5000 years). Superimposed on this long-term structure, both modes exhibit pronounced suborbital variability, indicating that millennial-scale fluctuations are an intrinsic feature of the system rather than being confined to intermediate climate states as defined by large-scale boundary conditions (i.e., CO_2_ level or GMSL), consistent with the ISOW_data_ stack.

**Fig. 2.**
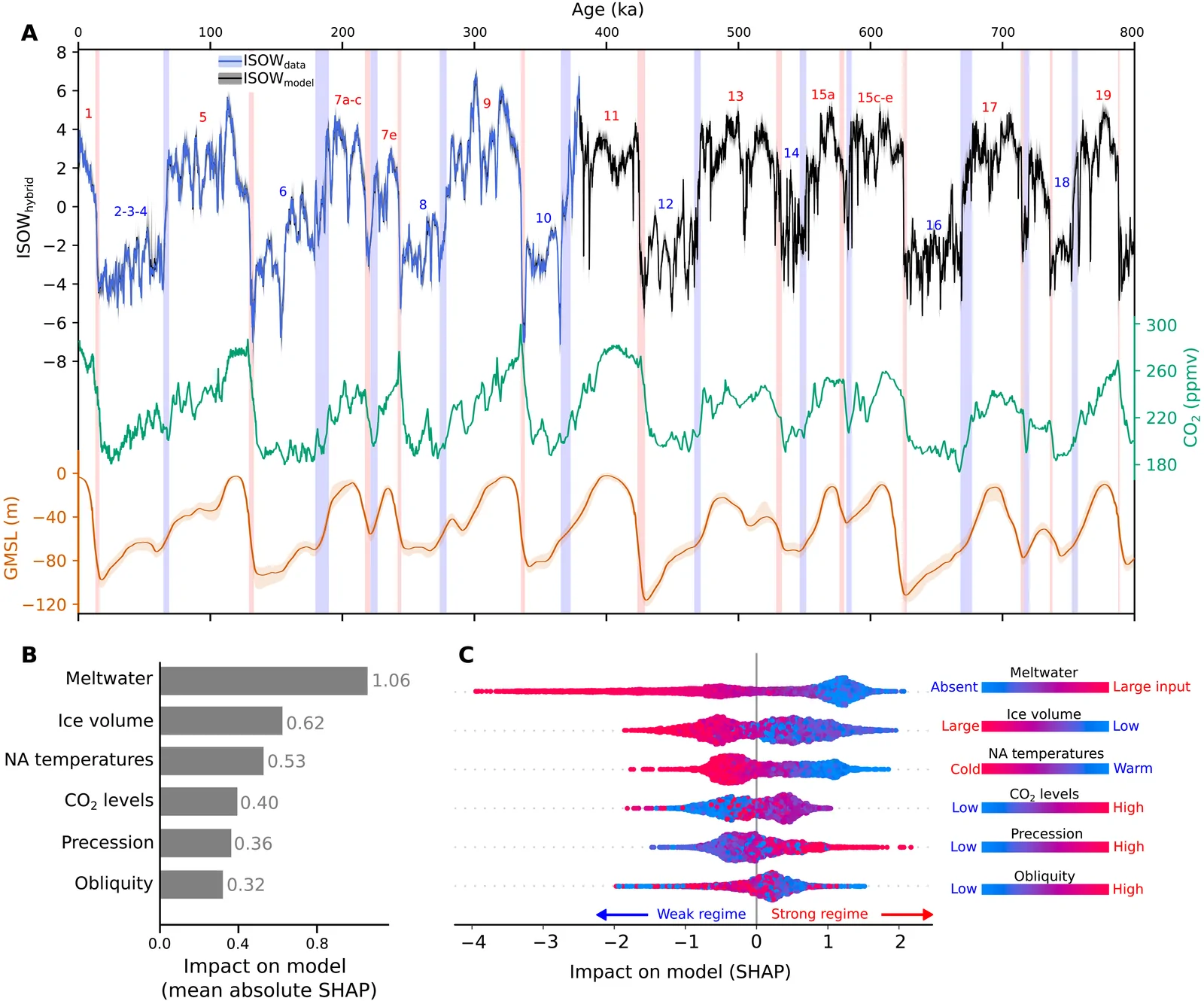
ISOW_hybrid_ strength stack over the past 800,000 years and model variable influences. (**A**) From top to bottom: combined ISOW strength records based on ISOW_data_ (blue) over the last 380 ka and ISOW_model_ (black) extending back to 800 ka, atmospheric CO_2_ composite record (*41*), and GMSL (*40*). Vertical bars indicate transitions from strong to weak modes (light blue) and weak to strong modes (light red). Numbers refer to Marine Isotope Stage numbers. (**B**) Relative importance of each input variable used in the predictive model. (**C**) Violin plots of SHAP values (impact on model’s predictions) for each climatic variable at any given time step used in the model. The colors indicate the variable value from low (blue) to high (red) values as they are used in the predictive model. NA, North Atlantic. 1 ka, 1000 years.

Attribution results (Fig. 2, B and C) reveal a clear hierarchy of influences. North Atlantic meltwater fluxes (IRD) and subpolar North Atlantic temperatures dominate ISOW variability, followed by large-scale boundary conditions such as ice volume and atmospheric CO_2_, and lastly orbital parameters. Meltwater fluxes strongly contribute to very weak states, whereas their near absence helps stabilize strong regimes, a pattern mirrored by ice volume (Fig. 2C). North Atlantic sea-surface temperatures and CO_2_ concentrations exert more symmetric influences, though with variable sign and amplitude (Fig. 2C). Orbital parameters have weaker but nonnegligible effects and appear to be necessary in the prediction on suborbital timescales (fig. S4; see the Supplementary Materials). Together, these patterns highlight that the ISOW strength’s variability arises from the combined influence of North Atlantic salinity and surface temperature forcing, ice volume, greenhouse gases, and orbital pacing.

### Forcing thresholds of ISOW regime shifts

To quantify the forcing conditions under which ISOW regime shifts occur, we identified persistent transitions between weak and strong ISOW states on orbital timescales directly from the ISOW_hybrid_ record (Fig. 3, A and B; see Materials and Methods). Each transition was mapped in the CO_2_-GMSL space, allowing us to delineate the boundary conditions associated with collapse and recovery events (Fig. 3C). The results reveal a notable asymmetry. Weak-to-strong transitions occur within a relatively broad range (∼242 to 265 ppm in CO_2_; −55 to −39 m in GMSL), whereas strong-to-weak transitions cluster within a narrower window of atmospheric CO_2_ and GMSL (∼201 to 208 ppm; −57 to −50 m). This asymmetry indicates a clear bifurcation in background conditions: the boundary conditions required to recover a vigorous circulation differ from those that trigger its weakening, even though these transitions occur under contrasting climate states. ISOW recovery is therefore possible under a wider range of forcings, whereas its weakening is confined to a more restricted window. These thresholds form windows rather than sharp values (Fig. 3C), consistent with the influence of multiple interacting climate drivers.

**Fig. 3.**
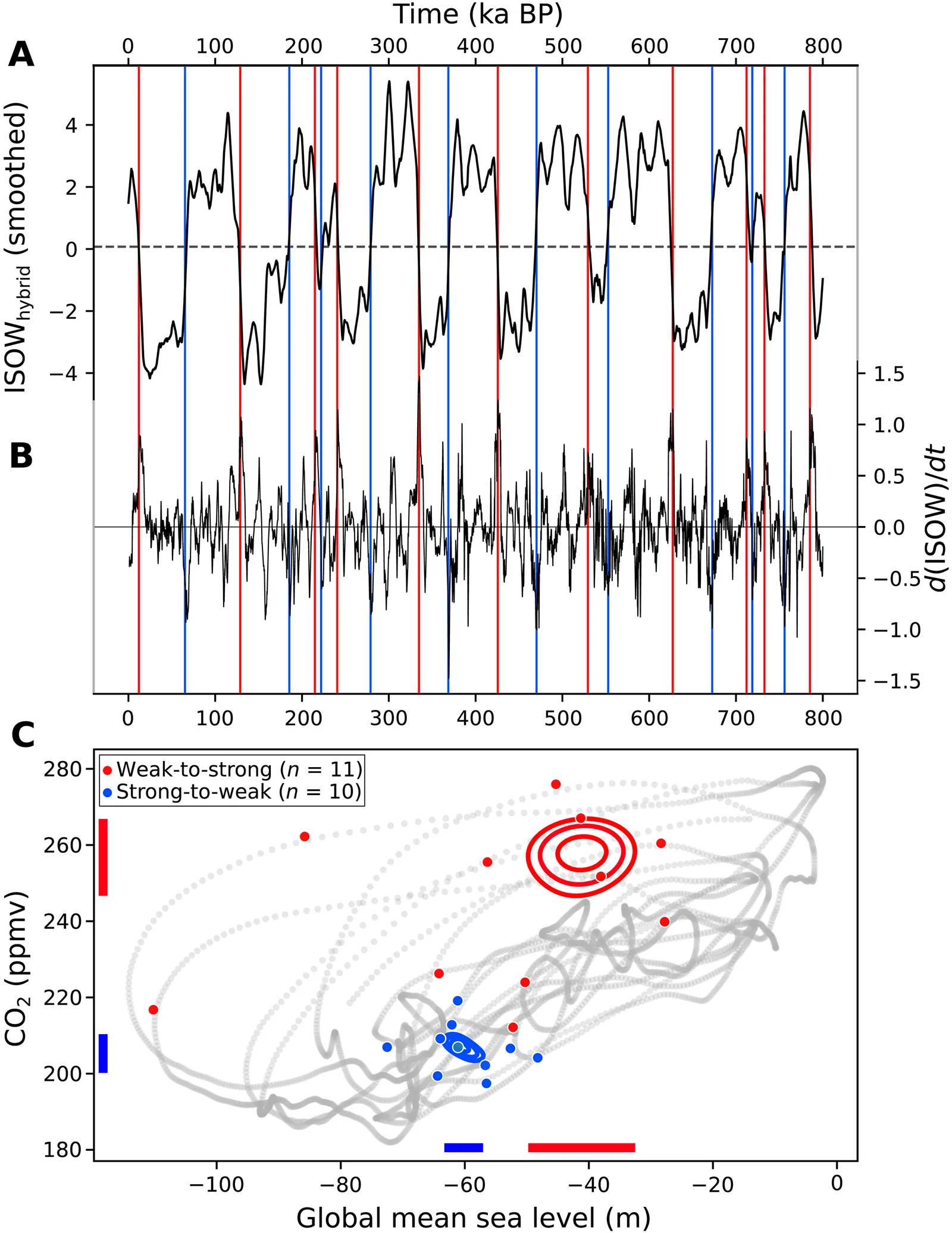
Threshold windows of ISOW regime shifts. (**A**) ISOW_hybrid_ (smoothed) with the regime separator (dashed horizontal line) and detected transitions (vertical bars; red = weak to strong, blue = strong to weak). (**B**) Time derivative *d*(ISOW)/*dt* showing the extrema used to time transitions. (**C**) CO_2_ and GMSL values at each transition summarized with 2D kernel density estimates. The 0.95, 0.97, and 0.99 KDE isodensity contours delineate high-density “threshold windows” (not probability coverage) for each direction (red = weak to strong, blue = strong to weak). These contour lines delineate the regions of highest transition density, interpreted as the “threshold windows” of the system. Horizontal and vertical bars in (C) are the 0.95 isodensity threshold windows for CO_2_ and GMSL, respectively (red = weak to strong, blue = strong to weak). All CO_2_-GMSL space combinations over the last 800,000 years are indicated in light gray in (C).

### Asymmetric loops on glacial-interglacial timescales

Having identified CO_2_ and GMSL windows for which ISOW transitions occurred, we now assess how their expression evolved through time. Across the last 800,000 years, ISOW_hybrid_ exhibits 23 regime shifts grouped into 11 glacial-interglacial cycles (Figs. 2A and 3A). Phase-space diagrams for each cycle (Fig. 4) reveal recurrent hysteresis loops: for identical values of atmospheric CO_2_ and GMSL, the ISOW strength can occupy two distinct states, reflecting the memory of the circulation’s history. A typical cycle is characterized by rising CO_2_ and GMSL while ISOW remains weak, until the weak-to-strong threshold is crossed. ISOW then persists in its strong mode until a later strong-to-weak transition closes the loop (Fig. 4).

**Fig. 4.**
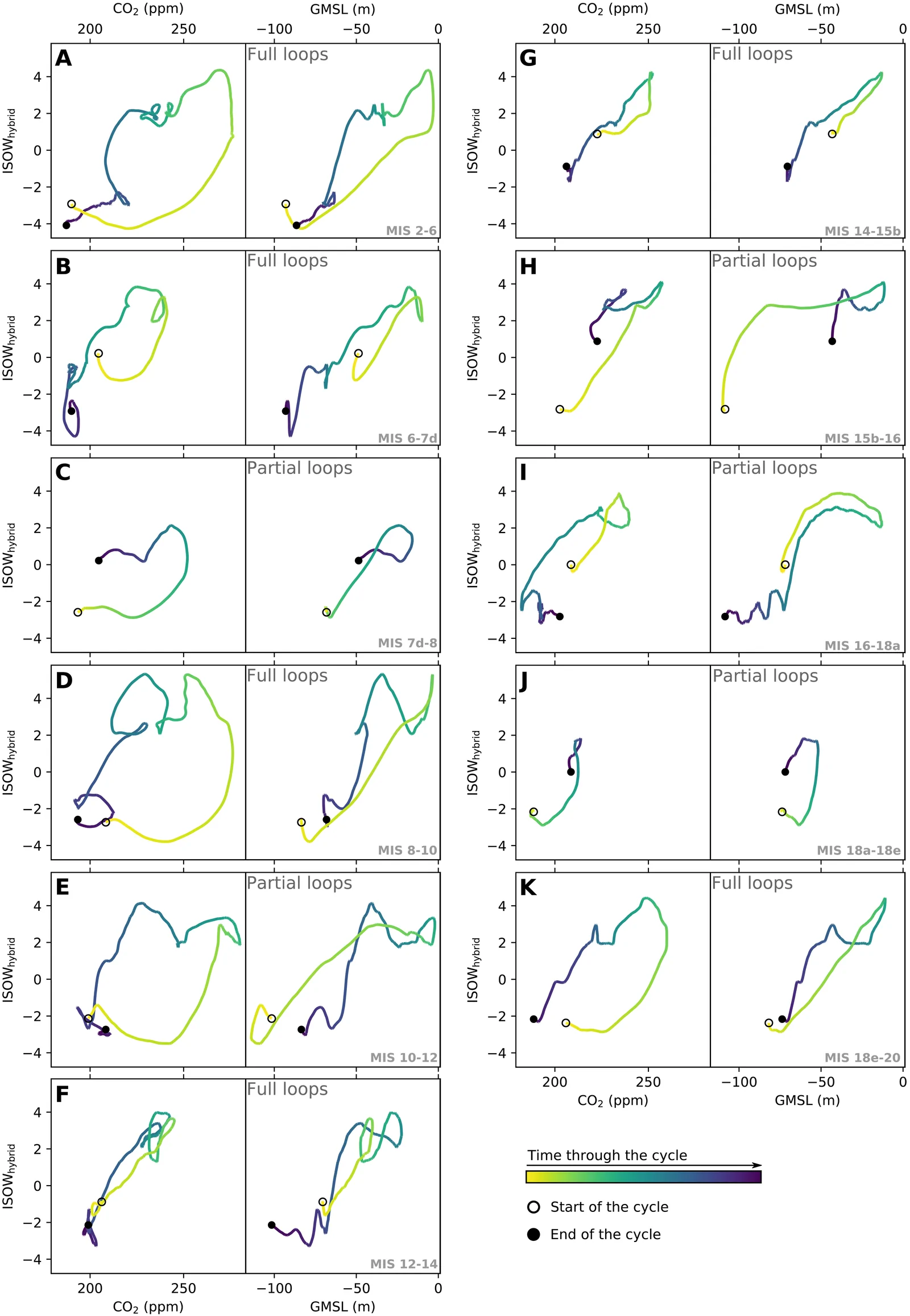
Phase-space dynamics of ISOW strength across glacial-interglacial cycles. (**A**) to (**K**) illustrate the smoothed ISOW_hybrid_ (7000-year smoothing window) evolution of a specific glacial-interglacial cycle. Each plot displays corresponding phase diagrams of ISOW_hybrid_ versus GMSL (right) and CO_2_ (left). The shaded colors indicate the temporal evolution, allowing the visualization of the phase relationship and cyclic behavior through glacial-interglacial transitions. The hysteresis loops highlight nonlinear transitions and delayed responses in the ISOW relative to ice volume and greenhouse forcing.

Detailed examination of individual cycles reveals two categories of hysteresis expression. Cycles spanning Marine Isotope Stages (MIS) 2 to 6, 6 to 7d, 8 to 10, 12 to 14, and 14 to 15b display full loops, with clearly separated collapse and recovery ISOW trajectories (Fig. 4). MIS 7d to 8, 10 to 12, 15b to 16, 16 to 18a, 18a to 18e and 18e to 19 show partial loops, where hysteresis is evident along one forcing axis (CO_2_ or the GMSL proxy) but not the other. These differences likely reflect variations in background climate state and forcing amplitude: Strong interglacials with warmer boundary conditions favor complete loops, whereas colder and shorter interglacials yield partial hysteresis of ISOW strength.

## DISCUSSION

### ISOW strength as a constraint on the deep North Atlantic circulation

At present, ISOW represents about a third of NADW (*9*, *10*). Whether ISOW variability can be used as a proxy for past changes in the strength of the deep Atlantic circulation remains debated, particularly under glacial conditions when deep-water formation may have partly reorganized toward lower latitudes (*12*, *48*–*52*).

Three independent lines of evidence indicate that ISOW_data_ instead tracks basin-scale changes in deep North Atlantic circulation rather than a purely local overflow signal. First, the two cores used in this study span a north-to-south transect along the Reykjanes Ridge at different water depths (∼2000 and ∼2800 m) and exhibit highly coherent variability across glacial-interglacial timescales, both in phase and in amplitude (*11*). This coherence provides a direct test of potential pathway effects. Modern observations suggest that approximately one-third of ISOW is diverted toward the Irminger Sea north of our southern site (*8*). If such a diversion had substantially and persistently increased under past climate states, one would expect a marked divergence between the northern and southern sites. No such divergence is observed. Furthermore, given the 100-year temporal resolution of our records and additional smoothing induced by bioturbation, the ISOW stack reflects a multicentennial integrated signal in which short-term fluctuations in pathway partitioning are effectively averaged out. The coherence between sites therefore reflects a persistent, large-scale common signal rather than transient routing effects. Second, a sediment provenance study demonstrates a persistent north-to-south transport, including during full glacial conditions (*11*), consistent with the continued activity of ISOW-derived flows rather than a replacement by waters formed further south. Third, ISOW_data_ exhibits coherent millennial-scale variability with the multisite NADW magnetic stack of (*36*), which integrates records from six sites along the NADW pathway from the Nordic Seas to the Bermuda Rise (fig. S5), with timing offsets not exceeding ∼800 years (Supplementary Materials). This coherence is further supported by a broad and consistent body of proxy evidence demonstrating that Nordic Seas’ convection and ISOW production remained active throughout glacial periods, including during full glacial conditions (*12*, *13*, *36*, *48*–*50*). As Kissel *et al.* (*36*) concluded, if additional convection sites contributed during glacial stages, they were either concurrently active with ISOW production or provided only a minor contribution to the waters exported southward. While we do not entirely exclude that convection sites further south may have partially contributed to NADW during glacials (*12*, *48*–*50*) due to a southward expansion of sea ice (*53*), the available evidence consistently points to ISOW variability as the dominant and coherent signal at our sites, closely coupled to basin-scale reorganizations of the deep Atlantic circulation.

No paleoclimate proxy provides a direct, spatially integrated measure of total AMOC transport, and both ISOW and ^231^Pa/^230^Th are best understood as qualitative, rather than strictly quantitative, indicators of past circulation strength. The ^231^Pa/^230^Th record from Bermuda Rise (*31*, *32*) is often treated as a benchmark for past AMOC strength, yet basin-wide compilations show that sedimentary ^231^Pa/^230^Th responses across the North Atlantic are strongly depth dependent, with shallow and deep sites frequently exhibiting different sensitivities to circulation changes (*30*, *33*, *34*, *54*). With a water depth of ∼4500 m, the Bermuda Rise record lies within this deep regime, where ^231^Pa/^230^Th more closely supports the conventional interpretation of enhanced southward ^231^Pa export under a vigorous overturning. However, the downstream (∼34°N) and integrative position (combining contributions from DSOW, Labrador Sea Water, and ISOW) of the Bermuda Rise site implies that its ^231^Pa/^230^Th signal is probably not linearly scaled with the total AMOC transport, and the interpretation of the Bermuda Rise record as evidence for a strong glacial AMOC remains debated (*30*, *33*–*35*, *54*–*56*). A direct comparison between ISOW_data_ and the Bermuda Rise record reveals a notable inverse correlation over their common interval (fig. S5), consistent with their opposite physical sensitivities, and both records reproduce comparable millennial-scale fluctuations. However, the amplitude of glacial states differs markedly: While ISOW_data_ defines a persistent and distinct weak mode, the Bermuda Rise record frequently returns to values close to interglacial levels during glacial periods, outside of massive iceberg discharge events (*32*). Rather than reflecting a true disagreement about AMOC strength, this contrast likely arises from the distinct and site-specific sensitivities of each proxy. In this context, our ISOW_hybrid_ stack and the Bermuda Rise record are best understood as complementary, qualitative constraints on different components of the deep circulation: ISOW_hybrid_ provides the first continuous, 800,000-year reconstruction of a major NADW branch at its source region, while the Bermuda Rise ^231^Pa/^230^Th record offers a downstream, basin-integrated, depth-specific perspective over the last ∼140,000 years.

These observations together indicate that the ISOW stack provides a robust constraint on the variability of Nordic Seas overflow and associated deep convection. While it should not be interpreted as a direct measure of total AMOC transport, its coherence with independent circulation records across multiple timescales indicates that these changes are closely linked to basin-scale reorganizations of the deep Atlantic circulation.

### Persistent ISOW bistability over the last 800,000 years

Both our data-only and hybrid stacks reveal a persistent alternation between two distinct circulation regimes of the deep North Atlantic over the past 800,000 years. In ISOW_data_, ISOW strength systematically clusters into a strong state during interglacials and a weak state during glacials, with rapid transitions between them and no evidence for long-lived intermediate plateaus (Fig. 1, A and E). The ISOW_hybrid_ reconstruction shows that this behavior extends back through the past 800,000 years (Fig. 2A): ISOW consistently occupies a strong or a weak mode, and regime shifts occur within a restricted window of atmospheric CO_2_ and GMSL (Fig. 3C). While CO_2_ and GMSL are not direct drivers of overflow strength, they act as integrative boundary conditions reflecting the combined influence of coupled processes, such as high-latitude heat exchange, wind forcing, or sea ice cover, that ultimately control hydrological changes, surface buoyancy, and deep convection in the North Atlantic and the Nordic Seas (*17*, *19*–*22*). The separation between collapse and recovery thresholds in forcing space, together with the presence of hysteresis loops in phase-space trajectories (Fig. 4), is a hallmark of bistable dynamics and supports the hypothesis that the deep Atlantic circulation admits multiple equilibria (*14*). In this framework, the two ISOW modes can be interpreted as distinct attractors, with glacial-interglacial forcing driving transitions between them rather than continuously modulating a single stable state.

Beyond the existence of two regimes, our results reveal that the deep Atlantic circulation undergoes abrupt reorganizations rather than smooth adjustments. The weak and strong ISOW modes are separated by sharp transitions, yet for most combinations of CO_2_ and GMSL, the circulation remains confined to one regime, implying that large excursions in external forcing are required to overcome the stability of each attractor. At the same time, the asymmetry between weak-to-strong and strong-to-weak transitions indicates that the underlying potential landscape is not symmetric, and that the pathway back to a vigorous circulation is dynamically faster than the pathway to its collapse. Together, the mode structure, threshold windows, and asymmetric loops point to a deep Atlantic circulation that is bistable on orbital timescales, with strong path dependence and memory of past states. In this sense, our reconstruction provides an observational counterpart to the multiple-equilibria behavior long predicted by Stommel’s box models (*14*) and hysteresis experiments in fully coupled climate models (*19*–*26*). Such simulations have largely explored millennial-scale responses to idealized perturbations, whereas extending them to orbital timescales under realistic forcing remains computationally challenging. Here, our data-driven stack fills a key gap by demonstrating that the bistable regime inferred from models also characterizes the long-term variability of the Mid-to-Late Pleistocene deep Atlantic circulation under natural forcing.

Superimposed on this bistable system, both stacks exhibit pervasive millennial-scale variability that is not confined to a particular regime. After removing the orbital component from ISOW_data_ (*43*), the residual signal reveals large-amplitude millennial fluctuations throughout the last four climate cycles (Fig. 1, B, F, and G), including during intervals when ISOW is firmly in either the weak or strong mode. ISOW_hybrid_ confirms that such suborbital variability is present across the entire 800,000-year record (Fig. 2A). This pattern is consistent with reconstructions of interglacial DSOW ventilation changes (*57*), and challenges the common assumption that such AMOC instability is confined to glacial or intermediate climate states (*22*, *32*, *58*, *59*). Instead, our results suggest that the deep North Atlantic circulation is best described as a bistable system with superimposed millennial-scale variability: orbital-scale forcing sets the existence and relative occupancy of two regimes, while internal and regional processes generate millennial-scale variability within each regime. In this sense, the stable modes of the Mid-to-Late Pleistocene deep North Atlantic circulation are not quiescent equilibria, but states that accommodate substantial intrinsic variability superimposed on their long-term structure.

### Mechanisms of ISOW regime shifts

The clustering of ISOW regime shifts around intermediate ice-sheet configurations (Fig. 3), which correspond to roughly half a typical glacial-interglacial GMSL change (∼−60 to −40 m), provides observational constraints on the boundary conditions under which overturning instability emerges. Similar threshold behavior has been identified in climate model sensitivity experiments (*20*–*25*). Consistent with these modeling studies, our results demonstrate that such thresholds operated repeatedly and coherently across multiple glacial-interglacial cycles in the natural system. This data-driven characterization of threshold location and width therefore offers a benchmark for evaluating climate models, not only in terms of whether they produce Nordic Seas overflow bistability, but also in how realistically they represent the range of forcing conditions over which regime shifts occur. In particular, the broader CO_2_ range associated with regime shifts in our reconstruction, extending up to ∼265 ppm, suggests that the instability domain of the real ocean-climate system is wider than that simulated in many idealized model experiments, likely reflecting the combined influence of multiple interacting forcings, feedbacks, or physical mechanisms.

The asymmetric threshold structure further implies distinct mechanisms operating along different branches of the hysteresis loop. Weakening under low CO_2_ concentrations and intermediate ice volumes is consistent with the expansion of sea ice over the Nordic Seas, which reduces air-sea heat exchange at high latitudes, freshens surface waters, and enhances upper-ocean stratification, thereby weakening deep convection (*19*, *60*, *61*). Once overturning weakens, the associated reduction in northward salt transport reinforces the low-density surface layer, locking the system into the weak mode through the salt-advection feedback (*14*, *16*, *26*). Strengthening, by contrast, is favored under higher CO_2_ and intermediate ice-sheet volume. During deglaciations, strong freshwater fluxes from northern ice-sheet melting (*62*) form a low-salinity surface layer that insulates Atlantic inflows and delays recovery despite increasing radiative forcing. As CO_2_ rises and sea ice retreats in the Nordic Seas (*63*), surface densities remain below the threshold required to trigger renewed deep convection until freshwater inputs decline and/or additional salt is supplied to the Atlantic surface system, for example, via Indian-Atlantic exchanges (*64*). Once this threshold is crossed, the salt-advection feedback rapidly locks the system into the strong mode. This sequence provides a physical explanation for the observed asymmetry, with weak-to-strong recoveries (on the order of ∼2000 years) occurring on average ∼2.5 times faster than subsequent strong-to-weak collapses (on the order of ∼5000 years). These two feedbacks act in opposite directions on the current circulation state: Sea ice and freshwater inputs stabilize it in the weak mode by reinforcing stratification, whereas salt and heat inputs destabilize it by promoting deep convection and triggering regime transitions.

### Evolving sensitivities of Nordic Seas overflow waters

The classification of hysteresis loops (Fig. 4) echoes the framework proposed by Yin and Berger (*65*, *66*), distinguishing pre- and post-MBE interglacials. Although pre-MBE interglacials are characterized by lower atmospheric CO_2_ concentrations (*41*) and, even if it is still debated, larger ice sheets (*40*, *67*, *68*), ISOW intensity itself shows no systematic change across the MBE (Fig. 2A). This classification (Fig. 4) does not reveal a systematic difference in loop completeness between pre- and post-MBE cycles. The narrower CO_2_ range spanned by pre-MBE loops reflects the reduced amplitude of glacial-interglacial CO_2_ variability at that time. We therefore further investigate this evolution by examining whether the phase relationship between ISOW_hybrid_ and external forcing changed across the MBE.

Spectral analyses reveal a persistent 100-ka pacing of ISOW variability (fig. S6), but with markedly different phase relationships before and after the MBE (Fig. 5, left panels). Over the full record, the 100-ka ISOW band is coherent (i.e., well correlated) with ice volume, CO_2_ concentrations, freshwater fluxes, and subpolar North Atlantic temperatures, yet the timing of its response evolves (Fig. 5, right panels). Before the MBE, ISOW tracked these boundary forcings almost synchronously: Transitions occurred nearly in phase with freshwater and ice volume changes, and with only short lags (∼5000 years) relative to CO_2_ and subpolar North Atlantic temperature forcings (Fig. 5). After the MBE, by contrast, the 100-ka ISOW band consistently lagged all four drivers by several thousand years (5000 to 10,000 years), indicating a delayed adjustment of the deep Atlantic circulation to external forcing. This systematic increase in response time demonstrates that the sensitivity of the deep ocean was reorganized across the MBE, consistent with the change in hysteresis loop geometry (Fig. 4).

**Fig. 5.**
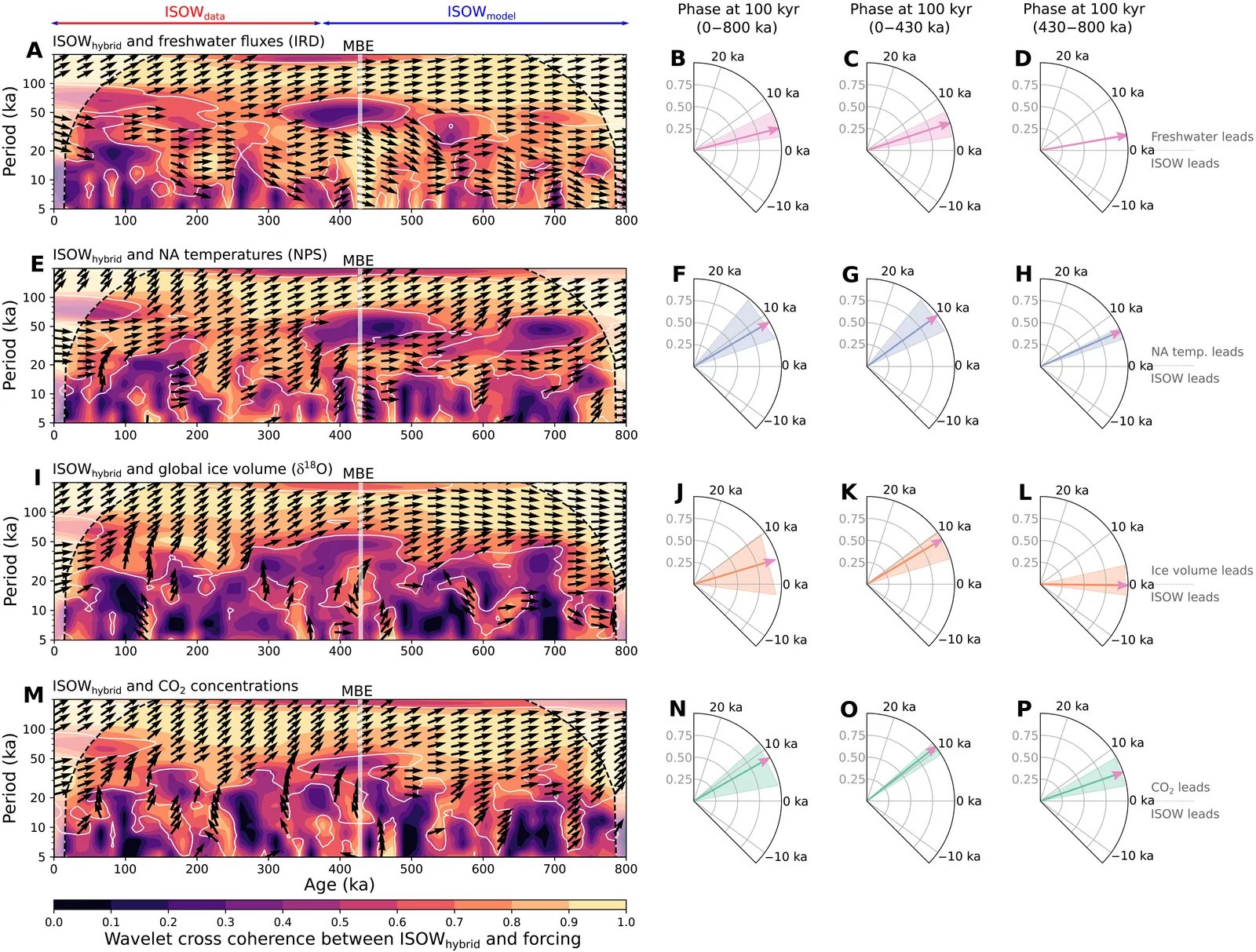
Coherence and phase relationships between ISOW variability and external forcing. Left panels show cross-wavelet coherence spectra between ISOW_hybrid_ and key climate drivers: freshwater fluxes (**A**), subpolar North Atlantic temperatures (**E**), global ice volume (δ^18^O) (**I**), and atmospheric CO_2_ concentrations (**M**). White contours indicate the 95% confidence interval, and warmer colors indicate higher coherence. Arrows represent the phase relationship between the two signals: Rightward arrows indicate in-phase variability, leftward arrows indicate anti-phase variability, while upward (downward) components indicate that ISOW lags (leads) the forcing. Right panels (**B** to **D**, **F** to **H**, **J** to **L**, and **N** to **P**) summarize the dominant phase relationships in the 100-ka band for the last 800 ka (B, F, J, and N), the post-MBE interval (C, G, K, and O), and the pre-MBE interval (D, H, L, and P). Together, these diagnostics highlight a persistent 100-ka pacing of ISOW strength, with systematic phase shifts across forcings and between pre- and post-MBE climate states.

The persistence of comparable interglacial ISOW strength before and after the MBE, despite higher CO_2_ concentrations during interglacial peaks and more pronounced orbital variability after ∼430,000 years ago (*41*, *46*), further indicates that the reorganization primarily affected the timing of overturning rather than its amplitude. Before the MBE, interglacial seasonal Arctic sea ice allowed Atlantic inflows to ventilate the Nordic Seas efficiently (*69*), promoting rapid transitions once thresholds were crossed. After the MBE, the establishment of Arctic perennial sea ice (*69*) and enhanced freshwater delivery to the Nordic Seas during deglaciations (*65*) increased surface stratification, delaying the resumption of Nordic Seas’ convection. At the same time, stronger Agulhas leakage (*70*) supplied additional salt to the Atlantic basin during deglaciations, which, together with reduced freshwater fluxes, eventually triggered deep-water strengthening through the salt-advection feedback (*64*), speeding up the return to the strong regime. A larger fraction of the Atlantic inflow may also have been diverted toward dense Arctic intermediate layers rather than directly feeding the overflows under high CO_2_ levels, consistent with modern observations (*71*). This redistribution of the Atlantic inflow provides a plausible explanation for why interglacial ISOW strength remained similar across the MBE despite a stronger climatic forcing. In this framework, the processes responsible for bistability remained the same as those identified in the previous section, but their relative influence changed: Post-MBE conditions enhanced the stabilizing role of sea ice and freshwater relative to the destabilizing effects of salt and heat, requiring external forcing to persist longer to overcome a more stratified background state.

Together, these results show that ISOW bistability persisted throughout the Mid-to-Late Pleistocene, but not as a fixed property of the Nordic Seas overflow system. Instead, its effective sensitivity and response time depended on the background climate state. Looking ahead, our findings shift the focus from whether the Nordic Seas’ overflow can undergo abrupt, hysteretic transitions to how its stability boundaries may respond to anthropogenic forcing. As atmospheric CO_2_ levels currently rise beyond the Pleistocene range, an open question is whether the convection will remain within the strong mode of a bistable regime, or whether its stability landscape will be fundamentally reshaped, potentially enabling collapse pathways suggested by climate model experiments (*72*).

## MATERIALS AND METHODS

### Construction of the ISOW_data_ strength stack

The paleoclimate records used to construct the ISOW_data_ stack are described in detail in (*11*). Here, we briefly summarize the key elements relevant to the present study. We selected two out of the three sites located on the eastern flank of the Reykjanes Ridge (fig. S1), along the present-day ISOW pathway (*3*, *8*). One core is located at intermediate water depths in the northern part of the ridge (∼2000 m; MD03-2685-84Cq) and the other at greater depth further south (∼2800 m; MD03-2673-74Cq). A third nearby core (MD03-2679-80Cq), collected at a similar depth to MD03-2685-84Cq, was excluded because it is influenced by local turbiditic events (*11*). The records from both sites faithfully indicate the variability of ISOW strength and a similar north to south variability, suggesting that both sites are affected by the same water mass (*11*). Therefore, we consider that changes in ISOW spreading pathways along the Reykjanes Ridge (*8*) do not substantially affect the southern site. The resulting ISOW_data_ stack therefore represents a north-south transect that integrates both intermediate and deep components of ISOW flow while minimizing site-specific effects. We retain the published age models for both cores, based on the AICC2012 chronology (*73*, *74*) and described in (*11*).

Proxies of ISOW strength were selected based on two criteria: high temporal resolution and continuous coverage over the last 380 ka, corresponding to the maximum age of core MD03-2673-74Cq. The selected magnetic proxies include the anhysteretic remanent magnetization (ARM)–to–isothermal remanent magnetization (IRM) ratio and the carbonate corrected magnetic susceptibility (κ). The elemental concentrations derived from calibrated x-ray fluorescence analyses include the zirconium (Zr)–to–Rubidium (Rb) ratio, the titanium (Ti)–to–potassium (K) ratio, and the carbonate-corrected concentrations of iron (Fe) and Ti (*11*). Variations in ARM/IRM and ln(Zr/Rb) ratios primarily reflect changes in grain size, whereas the ln(Ti/K) ratio, the low-field κ_carbonate-free_, and Fe and Ti concentrations track basalt-rich sediment input transported from the Iceland-Scotland sill to the study sites (*11*). Given the mean temporal resolution of most of the individual records (<100 years), all data were linearly interpolated every 100 years between 1 and 380 ka before stacking.

To extract the common ISOW signal while accounting for proxy-specific uncertainties and noise, we applied a linear dimensionality-reduction approach within a Monte Carlo framework. We generated 10,000 principal components analyses (PCAs), in which each proxy was randomly resampled within its analytical uncertainty envelope, normalized to zero mean and unit variance, and completed by imputing missing values with the mean of the corresponding record. This procedure was repeated independently for each Monte Carlo realization. The final ISOW_data_ stack is constructed from the ensemble of PC1 time series obtained across the 10,000 Monte Carlo iterations. The median and uncertainty envelope (all percentiles from the 1st to the 99th) are given by the distribution of all PC1 for each time step. In all cases, PC1 explains more than 70% of the total variance.

### Climate variable selection for modeling ISOW

To reconstruct ISOW variability beyond the observational interval and extend it to the last 800,000 years, we developed a hybrid statistical modeling framework combining linear regression and machine learning techniques. This approach requires the selection of climate variables that capture the large-scale boundary conditions and regional processes influencing overflow strength. The variability of ISOW, which forms one of the two deepest branches of NADW, is partly modulated by meltwater fluxes in the subpolar North Atlantic (*14*–*16*, *32*, *58*) and is closely linked to surface temperature changes in this region (*58*, *75*, *76*). Stevenard *et al.* (*11*) further demonstrated a strong correspondence between the ISOW strength and δ^18^O from benthic foraminifera in the deep North Atlantic. Because benthic δ^18^O primarily reflects global ice volume at first order, this relationship indicates that ISOW variability closely tracks glacial-interglacial cycles paced by ice-sheet growth and decay. However, this does not imply a direct causal control. While ice volume does not directly control overflow strength, it acts as an integrative boundary condition that reflects the combined influence of climate processes affecting surface buoyancy and deep convection (*20*). It therefore provides a physically meaningful predictor for ISOW variability within the modeling framework. As a major radiative forcing of the climate system, atmospheric CO_2_ concentrations may also influence ISOW strength indirectly on orbital to millennial timescales. Recent studies (*42*, *76*) suggest a link between atmospheric CO_2_ and AMOC strength, via a bipolar seesaw mechanism, in which CO_2_ increases are associated with a weak AMOC mode on millennial timescales. Because these climatic variables are themselves modulated by orbital forcing, orbital parameters such as obliquity and precession (modulated by eccentricity) are also expected to exert an indirect control on ISOW variability (*22*, *77*).

On the basis of these different relationships, we selected six climate variables to evaluate the model’s ability to reproduce ISOW variability over the last 380 ka (fig. S7). Given the importance of accurate and harmonized chronologies between all records, the selection is based on two criteria: high-resolution and consistent chronologies that are similar to the AICC2012 (*73*, *74*) of the ISOW_data_ stack. Therefore, the predictors include orbital parameters (obliquity and precession) (*46*), the atmospheric CO_2_ concentrations from EPICA Dome C ice core composite record (*41*, *42*), the benthic foraminifera δ^18^O record from the IODP site U1385 using the “hybrid” age scale (*45*, *78*), and IRD and *Neogloboquadrina pachyderma* (NPS) abundances from ODP site 983 (*44*, *74*), transferred onto the hybrid chronology following (*78*). The latter two records are used as indicators of freshwater fluxes and sea-surface temperature changes in the subpolar North Atlantic, respectively. The use of a benthic δ^18^O record rather than a reconstructed sea level record is primarily based on the fact that sea level reconstructions or modeling present age inconsistencies with respect to the AICC2012 (*73*, *74*) and we favored chronological consistency (harmonized chronologies) in the paleoclimate records we selected. The IRD record was log-transformed before modeling to account for its skewed distribution and to better capture relative variability in freshwater input, resulting in a stronger linear relationship with ISOW_data_ record (Pearson *r* from −0.20 to −0.57).

Four climatic variables (CO_2_, δ^18^O, IRD, and NPS; fig. S8) exhibit significant (*P* value < 0.01) linear relationships with the ISOW_data_ stack. However, because atmospheric CO_2_ is inversely correlated with AMOC strength on millennial to centennial timescales (*42*, *76*) and strongly colinear with δ^18^O (fig. S8), its direct inclusion in a purely linear regression would obscure their coupled and potentially nonlinear effects on deep ocean circulation. We therefore restricted the linear component of the hybrid model to IRD and NPS, which represent the most direct forcings of the North Atlantic. By contrast, the machine learning component was designed to capture more complex and nonlinear relationships. All variables, including CO_2_, δ^18^O, and orbital parameters, were thus provided to the ML model so that their combined and interacting effects could be evaluated. The linear predictors (IRD and NPS) were also included in the ML component, given their significant correlation (*P* value < 0.01) with other features (fig. S8) and their plausible joint influence with large-scale climate drivers, ensuring that both large-scale patterns and residual variability were represented.

### ISOW modeling over the past 800,000 years

Our modeling framework combines two complementary approaches: a linear regression model from the scikit-learn Python library (*79*) and an ML component based on a gradient-boosted decision tree ensemble (XGBoost Regressor) (*80*). This hybrid strategy allows us to capture both large-scale, interpretable relationships and higher-frequency nonlinear variability in ISOW strength over the past 380 ka. The linear model alone explains ∼40% of the variance in the ISOW_data_ stack, while the ML component captures residual structure not accounted for by the linear relationship (fig. S9).

To propagate uncertainties associated with the ISOW_data_ stack into the modeling framework, we applied a Monte Carlo procedure with 100 iterations. For each iteration, the target (ISOW_data_) time series was randomly drawn from a percentile of the ISOW_data_ uncertainty envelope, thereby incorporating reconstruction uncertainty into model training. The dataset was then randomly split into training (80%) and validation (20%) subsets, with missing values in the most recent interval (0 to 1 ka) filled using mean imputation. In each iteration, the linear regression model was first trained using ln(IRD) counts and NPS abundances as predictors. The residuals between the prediction and the original ISOW_data_ values were subsequently used as the target variable for the XGBoost model, which was trained using the full set of six selected climate predictors (fig. S9).

Hyperparameters for the XGBoost model were optimized independently for each Monte Carlo iteration using the Optuna framework (*81*). Once both the linear and ML components were trained, the combined model was applied to generate ISOW_model_ predictions over the full 0- to 800-ka interval (fig. S9). To interpret the contribution of individual predictors, we computed SHAP (*47*), an attribution method that quantifies the contribution of each variable to the model prediction at each time step. SHAP contributions were calculated from variable values exactly as provided to each model. Applied across the full dataset, SHAP values provide both the sign and magnitude of each feature’s influence on ISOW predictions, allowing us to assess the relative roles of individual climatic drivers within the multivariate framework. Because several predictors (e.g., CO_2_ and ice volume) are partially correlated, their contributions are evaluated conditionally within the model, and part of the explained variance may be shared among predictors.

### Threshold detection algorithm

We developed a fully data-driven algorithm to detect ISOW regime shifts directly from the ISOW_hybrid_ stack and to map their associated forcing conditions in CO_2_-GMSL space. External forcings were not used to define the timing of transitions. All time series (ISOW_hybrid_, CO_2_, and GMSL records) were first placed on a common, uniformly increasing age axis by linear interpolation at 1-ka resolution. To isolate orbital-scale variability, the ISOW_hybrid_ record was smoothed using a 7-ka moving-average window (*43*).

A separator “*S*” between weak and strong ISOW states was estimated from the distribution of the smoothed ISOW_hybrid_ values. By default, we fitted a two-component Gaussian mixture model to the smoothed distribution and defined *S* as the minimum of the mixture density between the two component means. Values below *S* were classified as weak states and values above *S* were classified as strong states. To suppress high-frequency state switching, state persistence was enforced using a majority rule within a 4-ka moving window.

Candidate regime shifts were identified as persistent crossings of separator *S*. For each crossing, we determined the transition time *t* as the point within a ±4-ka window that maximized the absolute time derivative of ISOW_hybrid_. The magnitude of each transition (ΔISOW) was calculated as the difference between mean ISOW_hybrid_ values of the post- and pretransition plateaus, computed over 4-ka windows (±4 ka around *t*) on either side of the transition. Transition direction (weak to strong or strong to weak) was assigned based on the sign of ΔISOW. To ensure robustness, we required transitions to exceed a local noise threshold, defined as the rolling standard deviation of the smoothed ISOW_hybrid_ record over a ±5-ka window on either side of the transition. Transitions with *|*ΔISOW*|* < 0.9 × σ (local standard deviation from the ±5-ka window) were discarded.

For each validated transition, atmospheric CO_2_ and GMSL values were extracted as the mean over a 3-ka window centered on *t*. Directional transition distributions were summarized using bivariate kernel density estimates (KDEs; Scott’s rule for bandwidth selection). Isodensity contours (0.95, 0.97, and 0.99; Fig. 3C) delineate the regions of highest transition density in CO_2_-GMSL space. These contours highlight where transitions most frequently occur but do not represent probability coverage. Reported threshold ranges in the text correspond to the extrema of the 0.95 isodensity projections.

To assess the sensitivity of the threshold detection to the smoothing method, we repeated the analysis using two alternative methods: (i) a cubic smoothing spline (UnivariateSpline *k* = 3) implemented in the SciPy Python library (*82*), and (ii) a low-pass filter with a cutoff at 1/12 ka^−1^ to remove millennial-scale variability (*39*). Threshold estimates derived from these methods are broadly consistent with those obtained using the moving-average filter (table S3), indicating that the choice of smoothing method does not substantially affect the inferred forcing windows.

### Spectral analysis

All spectral analyses were performed using the open source Pyleoclim Python library (*83*). Time series were linearly interpolated onto the ISOW_hybrid_ chronology and formatted as Pyleoclim “series” objects. Wavelet power spectra (fig. S6) were computed using the “wavelet” function, with significance assessed against an autoregressive red-noise background using the “ar1asym” method. Wavelet coherence analyses (Fig. 5) were performed using the “wavelet_coherence” function, with statistical significance evaluated using the “CN” method. Phase relationships shown in phase-wheel diagrams (Fig. 5) were derived directly from the wavelet coherence results. Across all records, wavelet spectra display significant power in the 100-ka band, whereas power in the obliquity (41 ka) and precession (19 to 23 ka) bands is more intermittent and less persistent through time (fig. S6).

## Supplementary Materials

### The PDF file includes:

Supplementary Text Figs. S1 to S12 Tables S1 to S3 Legends for data S1 and S2 References
