# Supplementary material for: Persistent bistability of Nordic Seas overflow waters over 800,000 years
Source: Sci Adv. 2026 Nov 4;12(45):eaee7886. doi: 10.1126/sciadv.aee7886 (PMC13733807; doi:10.1126/sciadv.aee7886)
Supplement: Supplementary file 1 [file sciadv.aee7886_sm.pdf]

Science Advances
AAAS

Supplementary Materials for

Persistent bistability of Nordic Seas overflow waters over 800,000 years

Nathan Stevenard *et al.*

Corresponding author: Nathan Stevenard, nathan.stevenard@uclouvain.be

Sci. Adv. **12**, eaee7886 (2026)
DOI: 10.1126/sciadv.aee7886

The PDF file includes:

Supplementary Text
Figs. S1 to S12
Tables S1 to S3
Legends for data S1 and S2
References

Other Supplementary Material for this manuscript includes the following:

Data S1 and S2

Supplementary Text

Assessment of the $ISOW_{data}$ stack

To validate our new stack, we compare it to the NADW stack produced by (*36*), which incorporates data from six different sites across the NADW pathways, including two within the ISOW flow pathway (**Fig. S1**). This reference stack is built from very high-resolution records that only cover the last glacial period between 20 and 60 ka. **Fig. S5** presents the comparison between the two stacks and shows that, despite different chronologies, they exhibit similar variability patterns during the last glacial period. The maximum offset between $ISOW_{data}$ and the NADW stack does not exceed ~800 years (**Fig. S5**). Both records capture most of the millennial-scale variability, although the $ISOW_{data}$ stack appears to be smoother on centennial to millennial timescales and resolves fewer Dansgaard-Oeschger events (*84*) than the glacial NADW stack (*36*). This difference likely reflects lower sedimentation rates at the southern core site during glacial periods (*11*), thereby reducing the resolution of glacial millennial-scale changes in the $ISOW_{data}$ stack. Furthermore, the NADW stack includes sites influenced by other deep water masses, such as the Denmark Strait Overflow Water (DSOW), and by the Nordic Seas' hydrography, potentially modulating the amplitude of millennial-scale events. Nonetheless, the $ISOW_{data}$ stack faithfully captures millennial-scale changes during the 20-60 ka period (**Fig. S5**), supporting its reliability over the entire 0-380 ka interval.

To further assess the large-scale significance of $ISOW_{data}$, we compare it to the high-resolution $^{231}Pa/^{230}Th$ record from Bermuda Rise (*31*-*32*) over the last ~140 ka (**Fig. S5**). Despite substantial differences in proxy definition, temporal resolution, and age uncertainties, the two records exhibit a significant inverse correlation (r = -0.61), consistent with their opposite physical sensitivities to deep Atlantic circulation strength. This agreement is notable given that the $ISOW_{data}$ stack integrates lower-resolution sedimentary records and is a "smoothed" signal due to the stacking procedure, whereas the Bermuda Rise record captures high-frequency variability at much finer resolution. At millennial scales, $ISOW_{data}$ reproduces most of the variability observed in the $^{231}Pa/^{230}Th$ record within chronological uncertainties, including major glacial fluctuations (**Fig. S5F**). However, the amplitude of these variations differs markedly between the two proxies. While $ISOW_{data}$ exhibits a well-defined weak (glacial) mode distinct from interglacial conditions, the

Bermuda Rise record frequently returns to values comparable to interglacial levels during glacial periods. This contrast suggests that the Bermuda Rise signal reflects not only changes in North Atlantic overflow strength, but also regional reorganizations in the structure and distribution of deep and intermediate water masses (*85*). Numerous studies, including those based on $^{231}Pa/^{230}Th$, indicate that the AMOC was generally weaker during the Last Glacial Maximum (LGM) or MIS 4 than during the last interglacial or the Holocene (*30, 33-35, 55-56*). The comparatively weak Bermuda Rise values (i.e. strong AMOC) during glacial intervals may therefore reflect a site-specific sensitivity to a persistent deep or intermediate circulation component, potentially involving contributions from water masses such as DSOW or LSW. In this framework, $ISOW_{data}$ captures the variability of a key overflow component of the deep circulation, while the Bermuda Rise record emphasizes regional expressions of basin-scale reorganizations.

Taken together, these comparisons indicate that, despite lower resolution and reduced sedimentation rates at the studied sites, $ISOW_{data}$ reliably captures the timing and structure of millennial-scale variability observed in independent reconstructions of deep Atlantic circulation. Although $ISOW_{data}$ should not be interpreted as a direct measure of total AMOC transport, it provides a robust and continuous constraint on the variability of Nordic Seas overflow and, more broadly, on changes in deep North Atlantic convection that actively contribute to AMOC variability across both glacial and interglacial climates.

Assessment of the $ISOW_{model}$ stack performance

The SHAP dependence plots (**Fig. S10**) highlight distinct patterns among predictors. Meltwater fluxes (IRD) and subpolar temperatures (NPS) show broadly similar responses, with SHAP values increasing as variable values decrease (**Fig. S10A, B**), confirming their role as primary surface controls on ISOW strength. Atmospheric CO_2 and benthic $\delta^{18}O$ also display comparable shapes (**Fig. S10C, D**), with strong positive contributions for intermediate values, but a decline toward interglacial CO_2 extremes, suggesting reduced sensitivity once boundary conditions are fully established (**Fig. S10C**). Orbital parameters exert weaker yet more nuanced influences (**Fig. S10E, F**). Precession shows little effect except at its extremes and around mid-range negative values (**Fig. S10E**), whereas obliquity exerts the largest control under low values, but with a reversal toward negative SHAP at very low obliquity, and diminished influence at high

obliquity (**Fig. S10F**). Together, these patterns underline the nonlinear and state-dependent role of both background climate and orbital forcing in modulating ISOW variability.

The model performance was assessed using 5-fold cross-validation (*79*). The entire dataset was split into five randomly subsampled datasets. The model is trained using four of them and validated using the remaining one. This operation is repeated five times to ensure the use of each fold as a validation dataset. We first trained the linear model and then computed the residuals. These residuals were then used to train the XGBoost model, which was subsequently evaluated on the validation set. Residuals were centered around zero in all folds (**Fig. S3**), with consistently high correlation coefficients (Pearson $r > 0.97$) and R^2 values above 0.94, indicating that the model captures both the amplitude and phasing of ISOW variability with strong robustness and no sign of overfitting (**Table S1**). To assess the impact of autocorrelation between neighboring data points in the training and validation datasets, we performed two additional sensitivity tests using contiguous time blocks of varying window sizes, split into 5 folds. This approach ensures that contiguous intervals are withheld from training, allowing us to evaluate whether the model can reproduce variability it has not been trained on. Results show a systematic decrease in performance metrics with increasing block size (**Table S2**). However, running $ISOW_{model}$ beyond 380 ka using 5-ka or 10-ka block-based training yields results nearly identical to the original approach, with only marginally increased noise and uncertainty (**Fig. S2**). Therefore, while the original training method is affected by autocorrelation that inflates performance metrics, all three approaches produce consistent reconstructions beyond the range of $ISOW_{data}$, suggesting that the model has robustly learned the underlying proxy-signal relationships and that its extrapolation skill is not an artifact of the validation design.

While the cross-validation shows the strong predictive skill of the hybrid model on known data, the main challenge is to evaluate the model performance for the interval between 380 and 800 ka, for which data of ISOW strength are sparse. Available ISOW reconstructions are limited to a few records from the North Atlantic, which provide complementary but heterogeneous or incomplete information on overflow strength. We therefore compared the $ISOW_{model}$ predictions to the proportion of non-cohesive (sortable) silt (SS%) record from ODP site 983 (*86*), covering the 750-800 ka interval, and the elemental ratio ln(Ti/K) tracer from IODP site U1314 (*39*), covering the last 1.1 million years.

As described in (*11, 87*), the $ISOW_{data}$ stack is mainly based on "ratio" proxies, which are more closely tied to the proportion of non-cohesive (sortable) silt (SS%) than to the mean grain size of the sortable fraction. Therefore, we compared the $ISOW_{model}$ predictions to the SS% record from ODP site 983 (*86*) (**Fig. S11**). The model successfully captures both long-term and millennial-scale variability, within age uncertainties of ~3 ka (**Fig. S11**).

On longer timescales, we compared the $ISOW_{model}$ variations to those of the ln(K/Ti) ratio from the IODP site U1314 (**Fig. S12**) (*39*). This proxy is a tracer of sediment provenance also used in constructing the $ISOW_{data}$ stack, based on the fact that Ti-rich basaltic particles originate from the Iceland-Scotland sill, while K-rich particles represent the typical terrigenous input in the North Atlantic. Site U1314, which is located close to core MD03-2673-74Cq (used to produce $ISOW_{data}$), also lies along the ISOW pathway, but suffers from low sedimentation rates during glacial periods, which reduces its ability to capture millennial-scale variability during these periods. Despite this limitation, the comparison with $ISOW_{model}$ shows a strong consistency on orbital timescales (**Fig. S12**). Both records display the same succession of glacial-interglacial cycles, and discrepancies during glacial periods are of the same order as those observed during the data-constrained 0-380 ka interval. Timing mismatches between records likely reflect chronological uncertainties inherent to heterogeneous chronological approaches, rather than deficiencies of the model itself. This comparison therefore supports the robustness of $ISOW_{model}$ back to 800 ka on glacial-interglacial timescales, while emphasizing the challenge of benchmarking modeled circulation strength against single-site sedimentary cores.

Sensitivity tests on climate feature combinations

To assess the relative contribution of each climatic predictor to the robustness of $ISOW_{model}$, we performed a series of sensitivity experiments using fixed inputs (IRD and NPS records) and varying combinations of benthic $\delta^{18}O$, atmospheric CO_2 concentrations and orbital parameters (obliquity and precession). In each configuration, the model structure remained hybrid (using both a linear and XGBoost Regressor model on residuals), and predictions were extended beyond the 0-380 ka training interval.

Starting from a minimal setup with IRD+NPS+orb (orbital parameters; **Fig. S4A**), the model captures the timing of glacial-interglacial cycles, but underestimates the transition sharpness and shows high-frequency noise. Removing orbital forcing and adding both benthic $\delta^{18}O$ and CO_2

concentrations (IRD+NPS+CO_2+$\delta^{18}O$; **Fig. S4B**) increases the amplitude of orbital-scale variability and reduces noise, but dampens suborbital-scale (<20 ka) variability. Re-introducing orbital forcing while dropping the benthic $\delta^{18}O$ (IRD+NPS+CO_2+orb; **Fig. S4C**) yields similar orbital-scale skill and better resolves suborbital variability. Swapping the CO_2 concentrations for benthic $\delta^{18}O$ (IRD+NPS+$\delta^{18}O$+orb; **Fig. S4D**) leaves the large-scale pacing essentially unchanged relative to **Fig. S4C**, but reduces suborbital amplitudes. The full model including all predictors (Fig. **S4E**) performs best overall, reconciling glacial-interglacial amplitude with suborbital variability and reducing noise.

Taken together, these tests show that CO_2 or $\delta^{18}O$ are required to recover the full amplitude of glacial-interglacial variability, whereas orbital forcing, especially in combination with CO_2, helps pace suborbital variability. Although orbital parameters have a lower standalone importance, their interaction with CO_2 or $\delta^{18}O$ measurably improves the model's skill (comparing **Fig. S4C-D** and **Fig. S4B**), indicating that they act primarily as modulators rather than primary drivers. The full predictor set therefore provides a compact and physically consistent reconstruction, with complementary roles for surface forcing (IRD, NPS), ice volume ($\delta^{18}O$), greenhouse gases (CO_2), and orbital pacing.

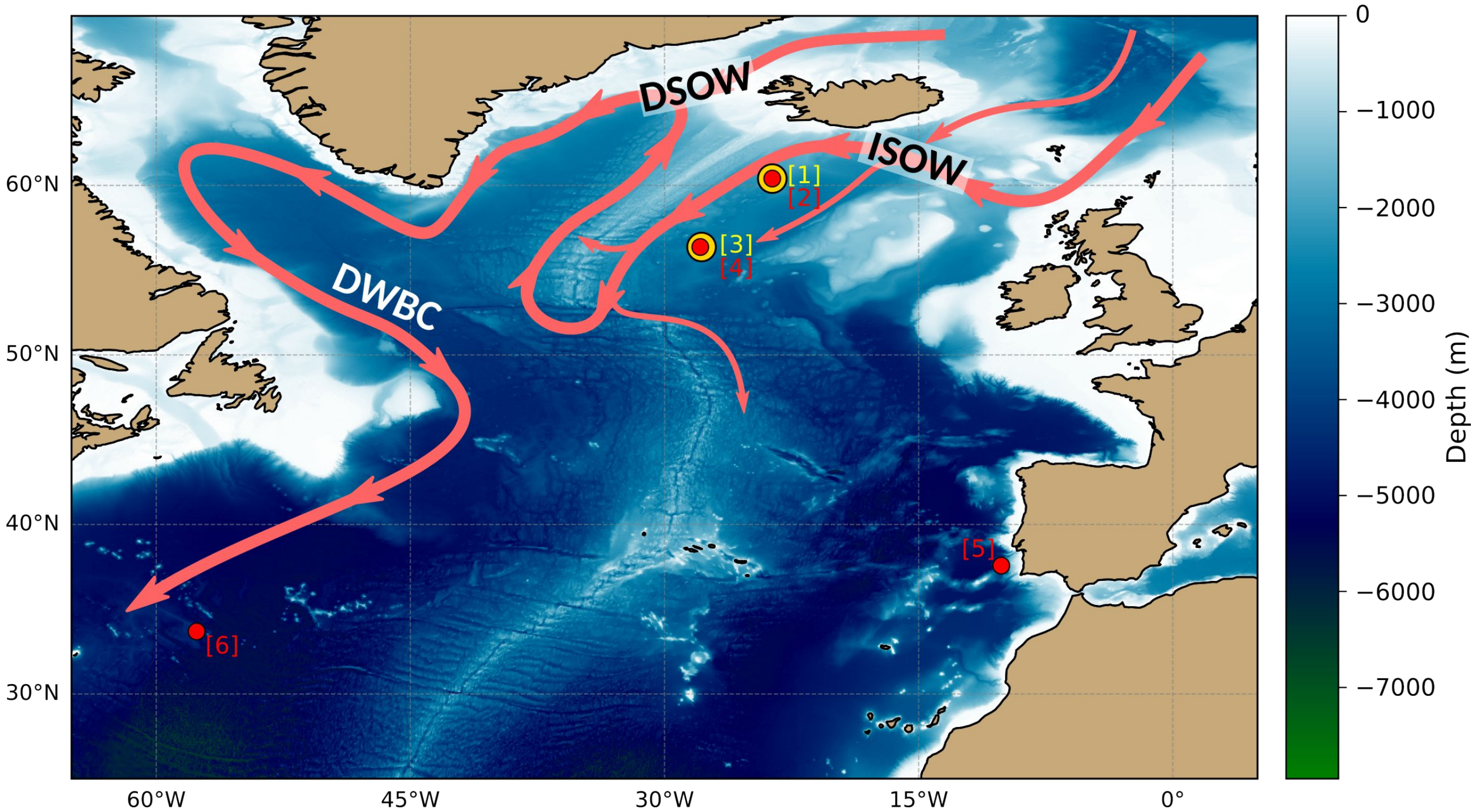

Fig. S1.

Nordic Seas overflow water pathways and study sites. Bathymetry is shaded in blue (darker = deeper). Arrows depict schematic pathways of the Denmark Strait Overflow Water (DSOW), the Iceland-Scotland Overflow Water (ISOW), and their continuation into the Deep Western Boundary Current (DWBC). Yellow circles locate the two core sites used in this study: [1] MD03-2685-84Cq (~2,000 m water depth) and [3] MD03-2673-74Cq (~2,800 m water depth). Red circles locate [2] ODP site 983, [4] IODP site U1314, [5] IODP site U1385 (Shackleton site), and [6] ODP site 1063 (Bermuda Rise). Arrows indicate qualitative routes based on (*3, 5, 8*).

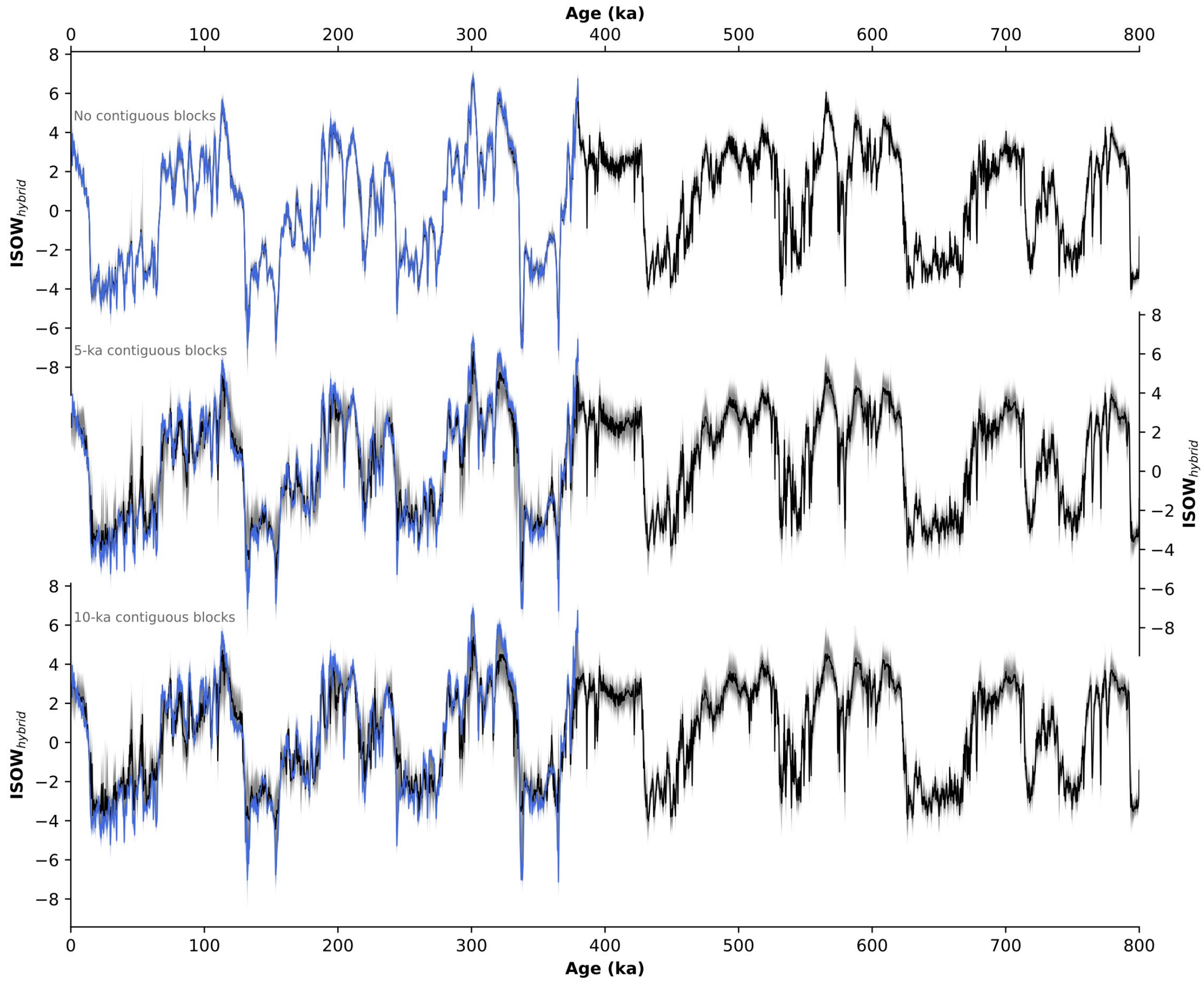

Fig. S2.

Comparison between the ISOW$_{model}$ stack with different block-based training methods. (Top) ISOW$_{model}$ without contiguous blocks (as used in this study); (middle) ISOW$_{model}$ trained with 5-ka contiguous blocks; (bottom) ISOW$_{model}$ trained with 10-ka contiguous blocks. Blue curves and shaded uncertainties represent the ISOW$_{data}$ stack, while black curves and shaded uncertainties represent the different ISOW$_{model}$ stacks.

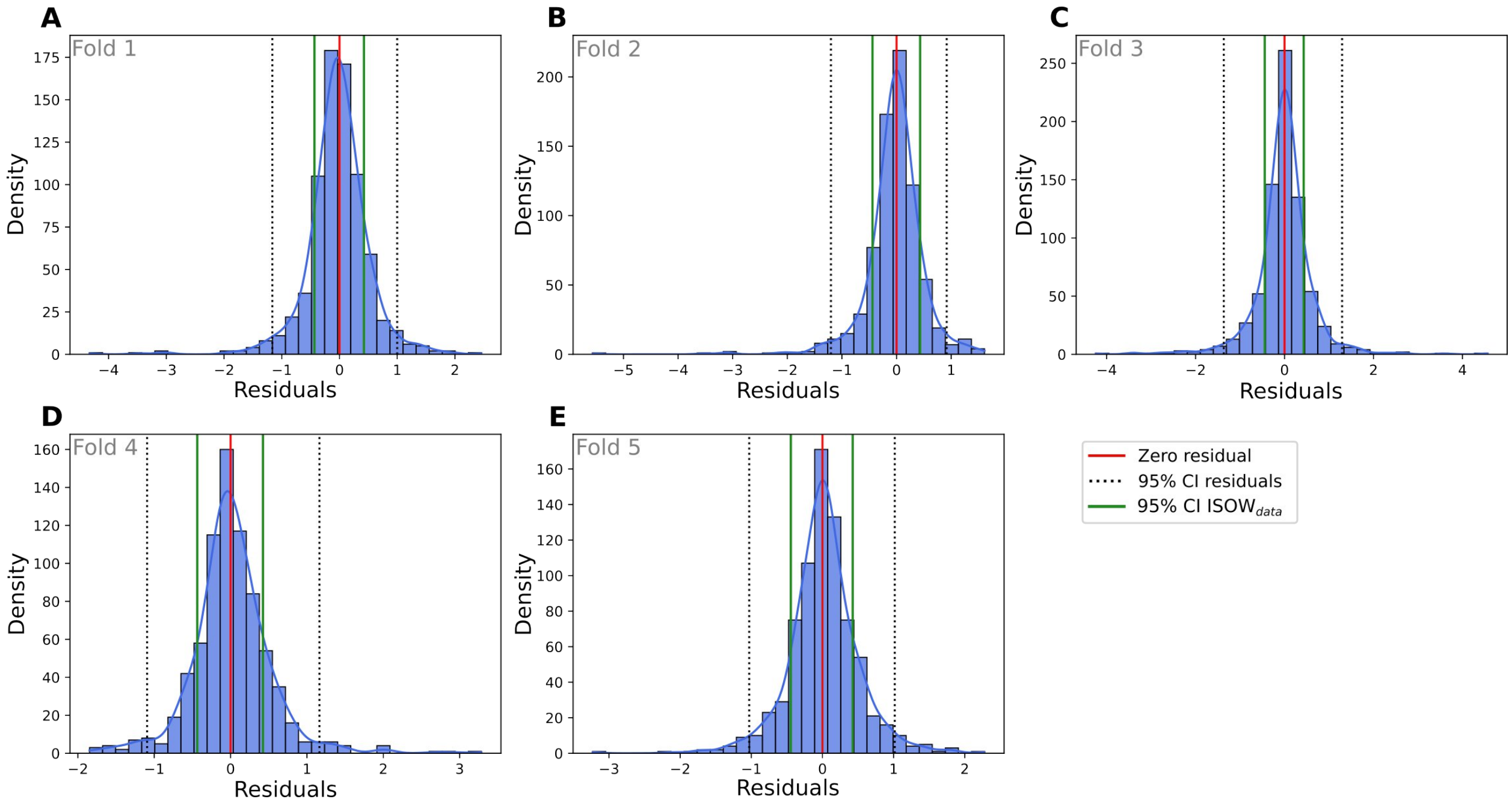

Fig. S3.

Cross-validation residual distributions of the hybrid model. Histogram and kernel density estimation of model residuals for each of the 5 cross-validation folds (ensuring 80% of the data are for training and 20% are for validation). Most residuals fall into the ISOW$_{\text{data}}$ uncertainty range (from the validation dataset). Residuals are approximately centered around zero in all folds, indicating unbiased predictions. The consistent shape across folds suggests stable model performance and no overfitting. Red lines denote the zero-residual axis, green lines denote the 95% confidence interval (CI) of ISOW$_{\text{data}}$, and dotted black lines are related to the 95% CI of the residual distribution for each fold.

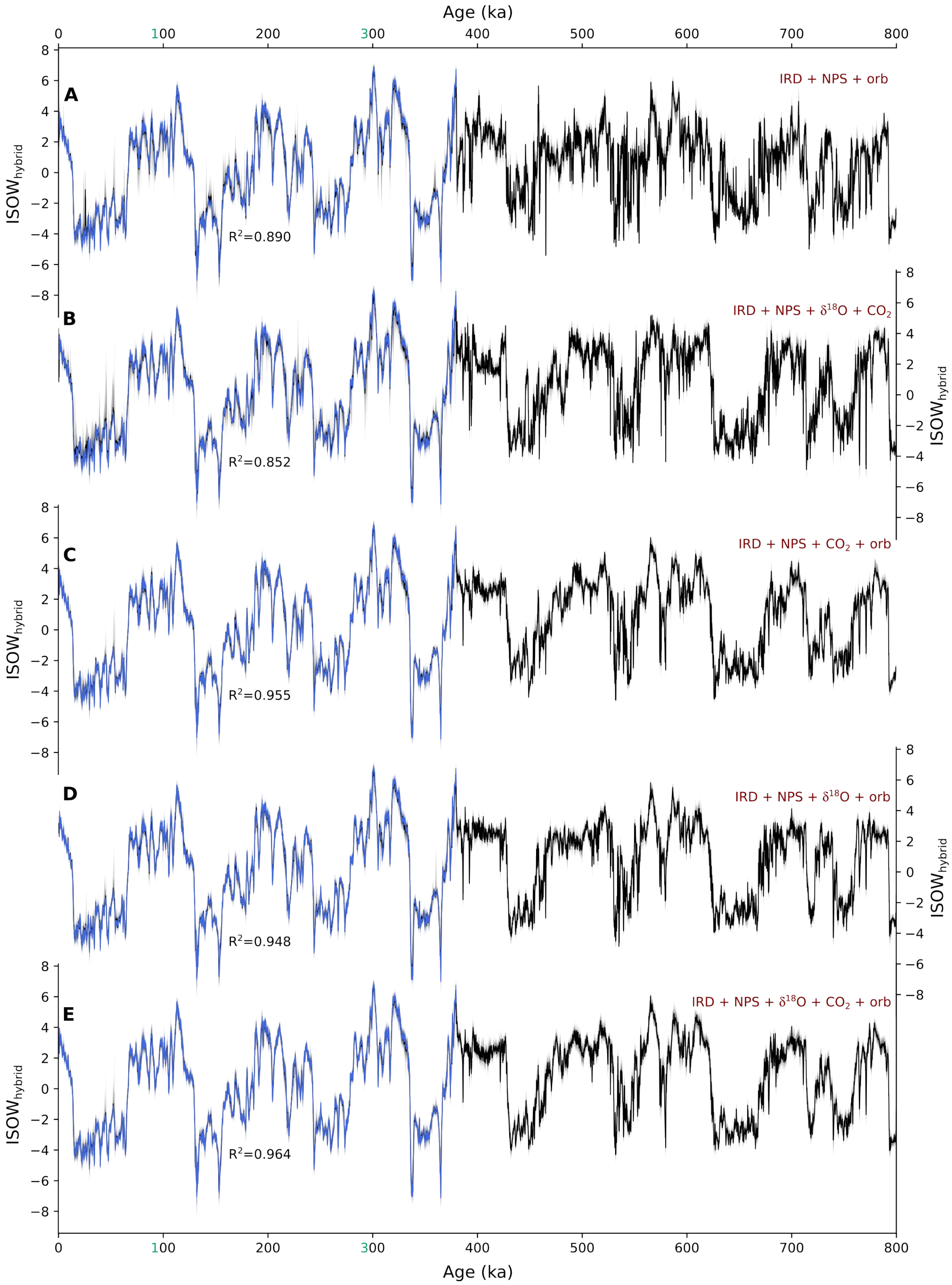

Fig. S4.

Time series of $ISOW_{model}$ based on different combinations of climate forcing inputs. Each panel shows $ISOW_{model}$ outputs over the last 800 ka (blue), with the calibration interval (0-380 ka) illustrated by $ISOW_{data}$ (red). In each panel, we successively change one or two predictors; with IRD, NPS and orbital parameters (**A**), with IRD, NPS, CO_2 concentrations and benthic $\delta^{18}O$ (**B**), with IRD, NPS, CO_2 concentrations and orbital parameters (**C**); with IRD, NPS, benthic $\delta^{18}O$ and orbital parameters (**D**); and with all features (the final $ISOW_{model}$ stack used in the main text) (**E**). While all models reproduce the general pacing of glacial-interglacial transitions, the full model (**E**) achieves the best performance (R^2).

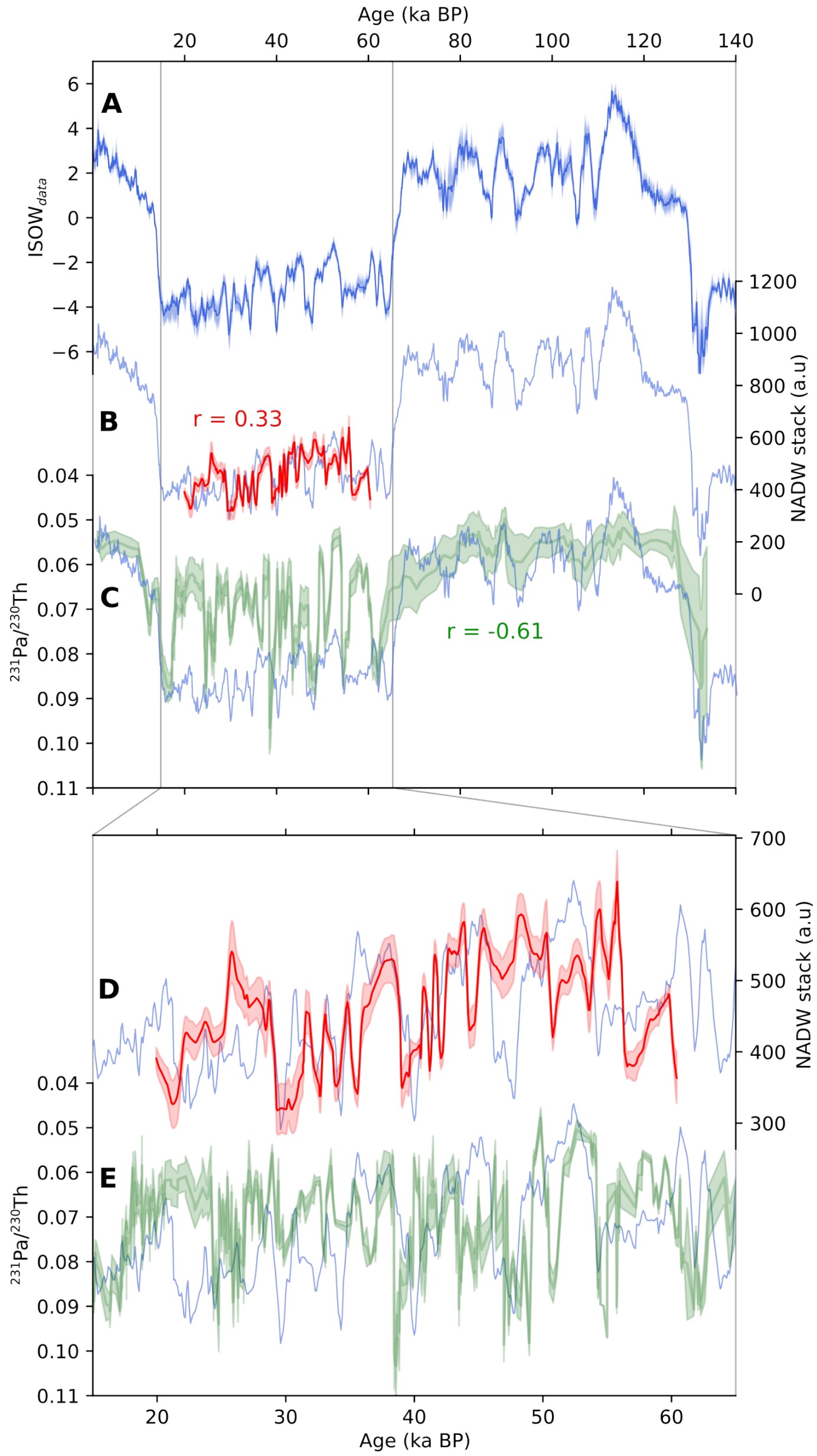

Fig. S5.

Comparison between DWBC records and ISOW$_{data}$ stack (this study) over the last 140 ka (top panel), with a zoom (bottom panel) on the period between 15 and 65 ka. (**A**) ISOW$_{data}$ stack on AICC2012 chronology; (**B**, **E**) NADW stack (*36*) on GICC05 chronology; (**C**, **E**) 5-point moving average of the Bermuda Rise ^{231}Pa/^{230}Th record from (*31, 32*). (**D-E**) Zoom over the 15-65 ka. Light blue curves in **B-E** are the median values from ISOW$_{data}$. Note that ISOW$_{data}$ records in **D-E** are rescaled to fit with the amplitude of the NADW and the ^{231}Pa/^{230}Th records.

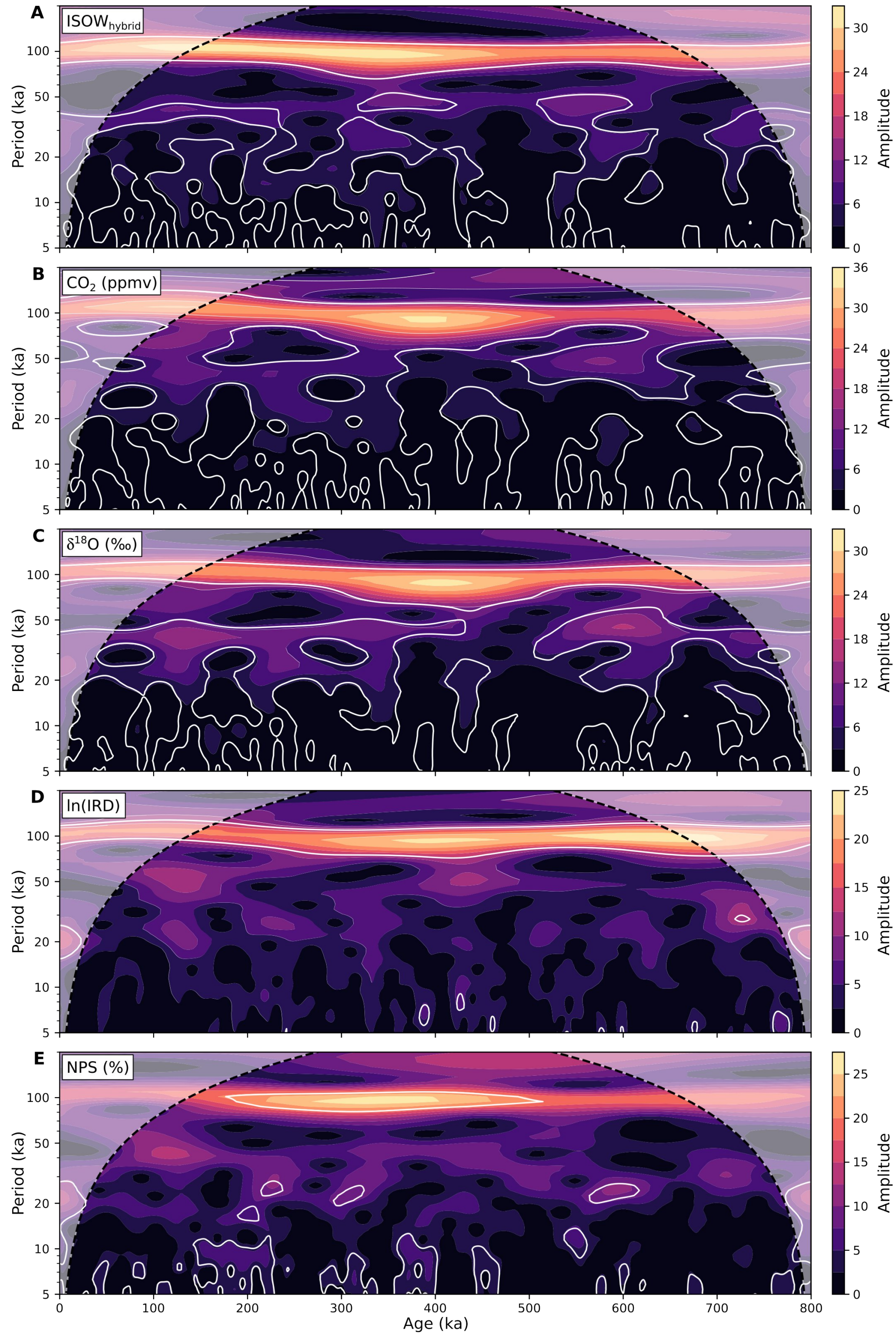

Fig. S6.

Spectral wavelet analysis of the ISOW$_{hybrid}$ and climate forcings. Wavelet analysis of (**A**) ISOW$_{hybrid}$, (**B**) atmospheric CO_2 concentrations, (**C**) $\delta^{18}O$ as ice volume change record, (**D**) IRD (log) as meltwater flux record, and (**E**) NPS as North Atlantic surface temperature record. The amplitude of power scale is different for each subplot. White contours represent the 95% confidence level.

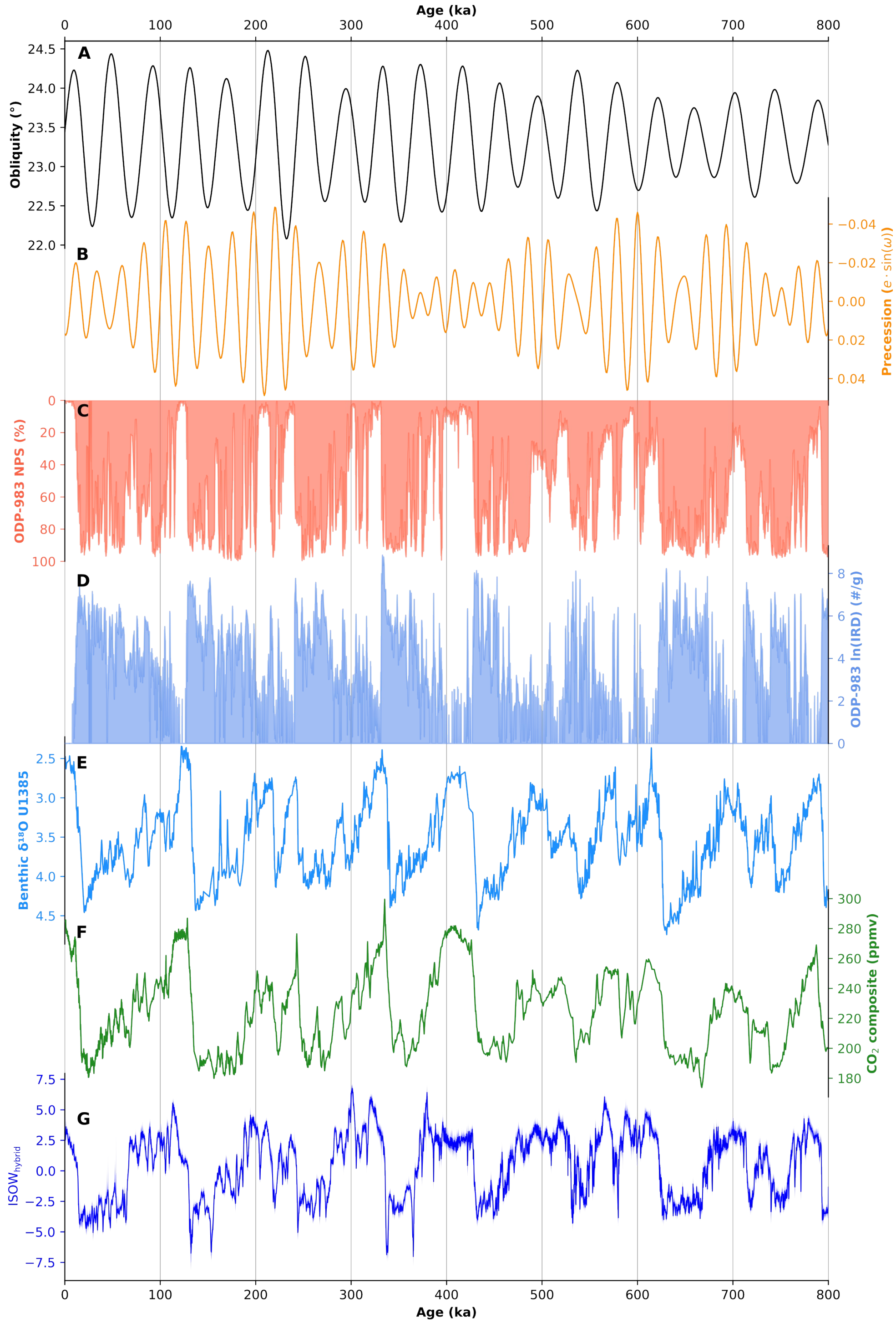

Fig. S7.

Climatic variables used in the model framework alongside with ISOW$_{\mathbf{model}}$. (**A**) Obliquity and (**B**) precession parameters (*46*), (**C**) N. pachyderma (NPS) and (**D**) log-transformed Ice-Rafted Debris (IRD) counts from ODP site 983 (*44*), (**E**) benthic $\delta^{18}O$ from IODP site U1385 (*45*), (**F**) atmospheric CO_2 composite record (*41-42*) and (**G**) ISOW$_{hybrid}$ stack (this study).

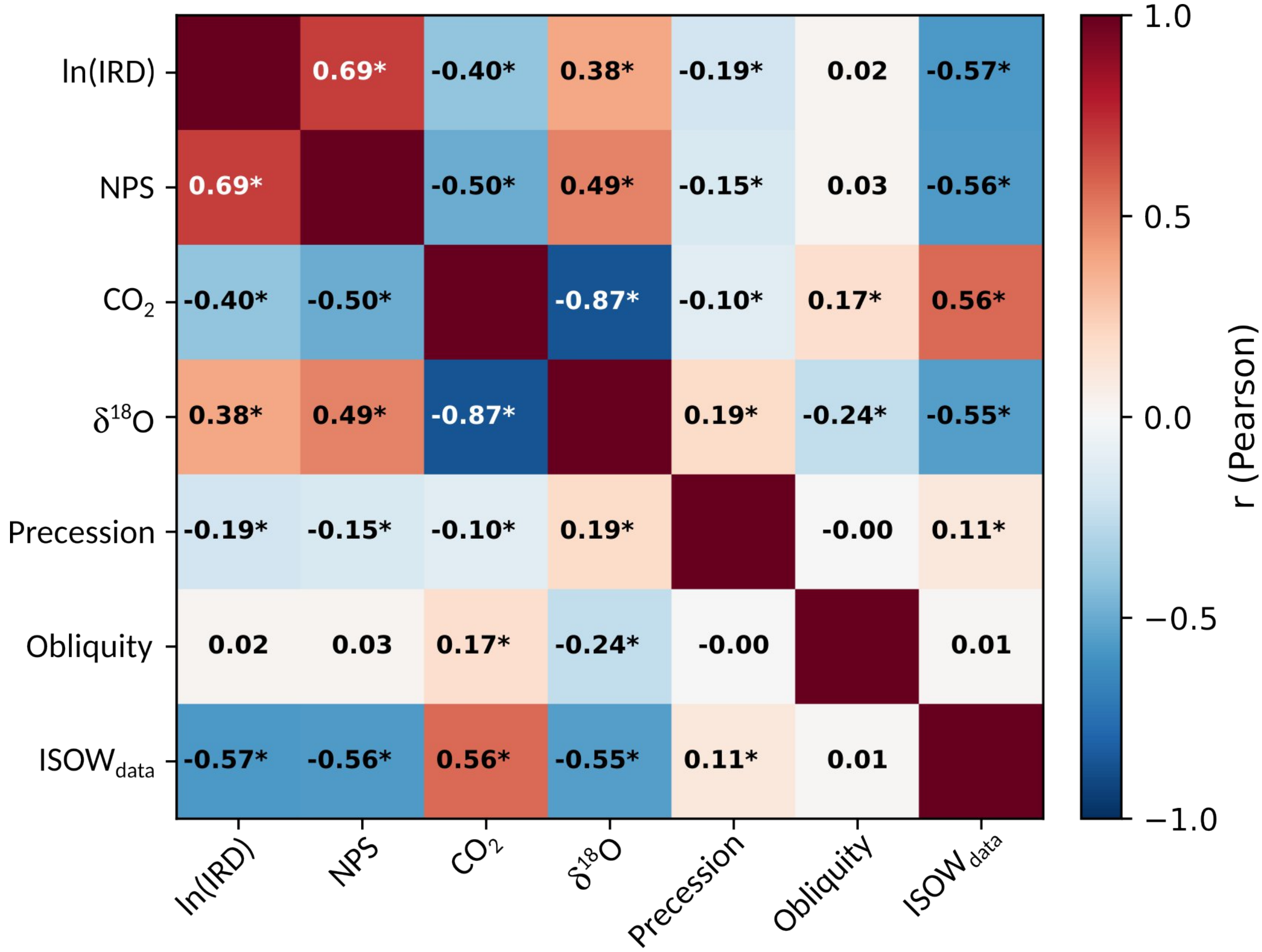

Fig. S8.

Matrix of correlation (Pearson) including the climatic variables and the $ISOW_{data}$ variability. Red indicates strong positive correlations, blue indicates strong negative correlations. "*" indicate significant correlations with *p-value* < 0.01.

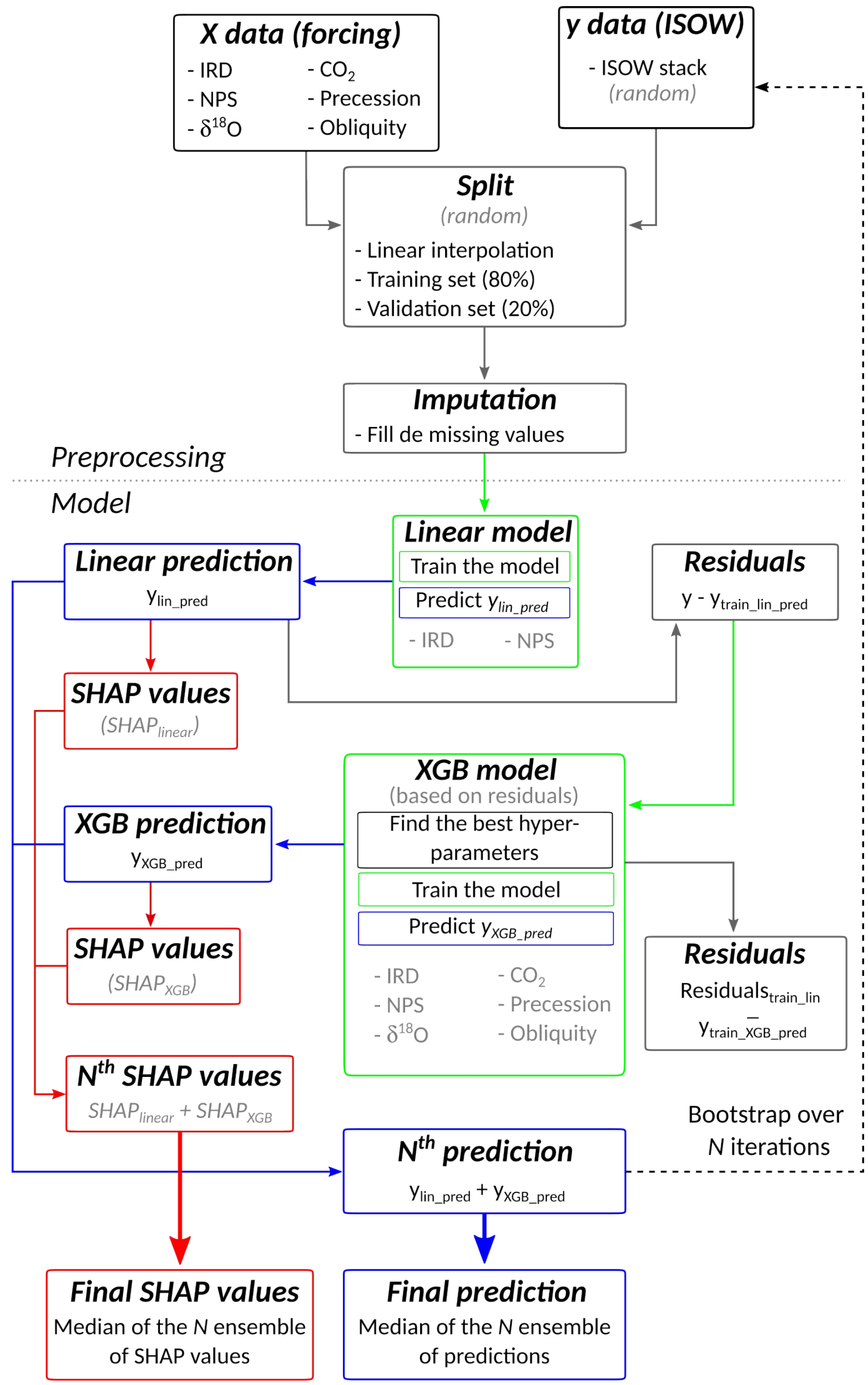

Fig. S9.

Schematic workflow of the two-step modeling and attribution approach. The target variable is randomly split into training and test sets. A first linear model is trained and residuals of this training are extracted. These residuals are used to train a second gradient boosting model (XGB). SHAP values are computed separately for both models and summed to estimate each variable's total contribution.

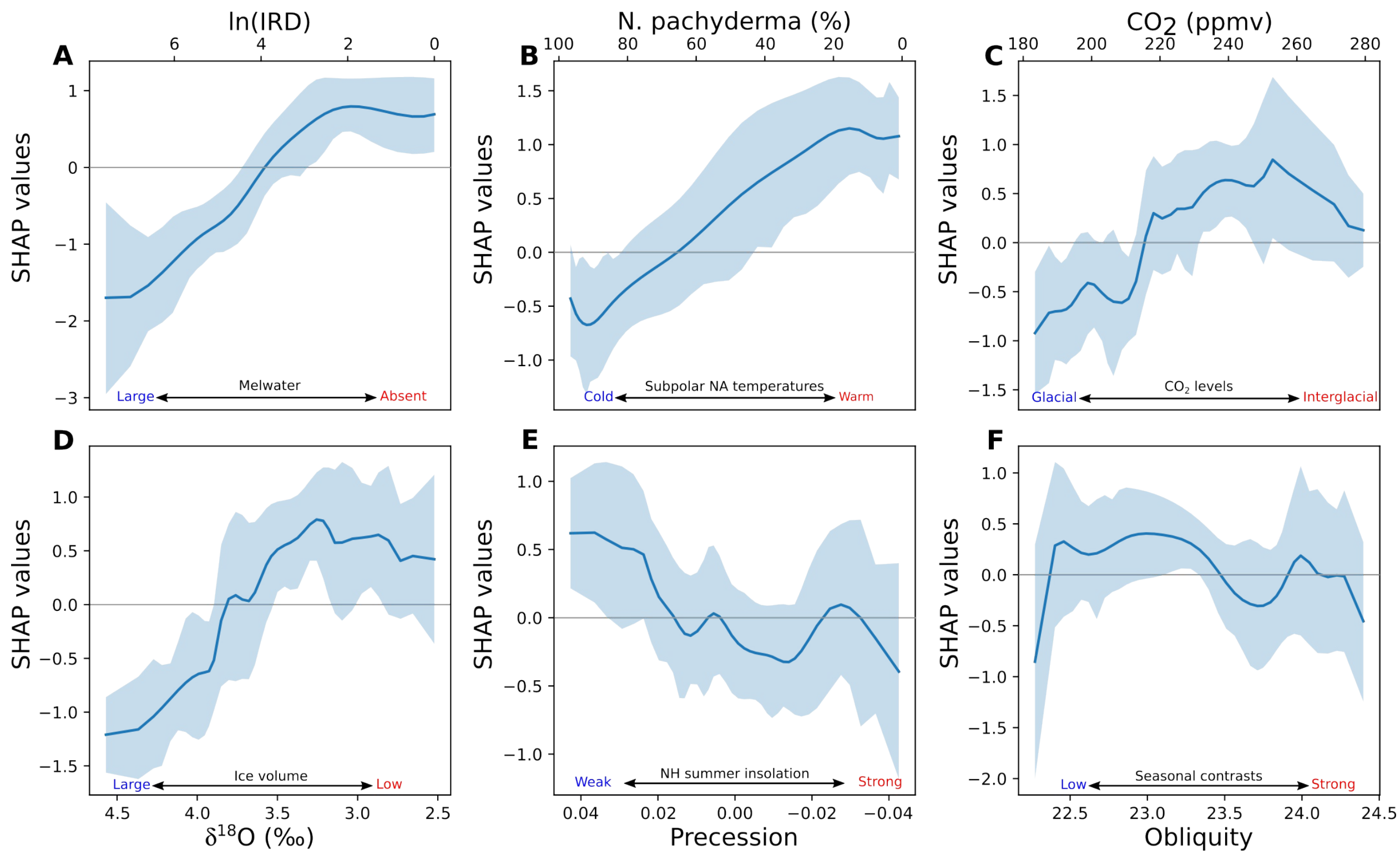

Fig. S10.

SHAP dependence bands for the hybrid model features. (**A**) Meltwater indicator (ln(IRD)), (**B**) subpolar North Atlantic temperature proxy (NPS%), (**C**) atmospheric CO_2 concentrations (ppmv), (**D**) ice-volume proxy (benthic foraminifera $\delta^{18}O$), (**E**) precession, and (**F**) obliquity. For each predictor (x-axis), SHAP values (y-axis) are summarized as a central trend (solid line: median SHAP across quantile bins of the predictor) and an uncertainty band (± 2σ, within-bin standard deviation). Curves are lightly smoothed with a cubic spline.

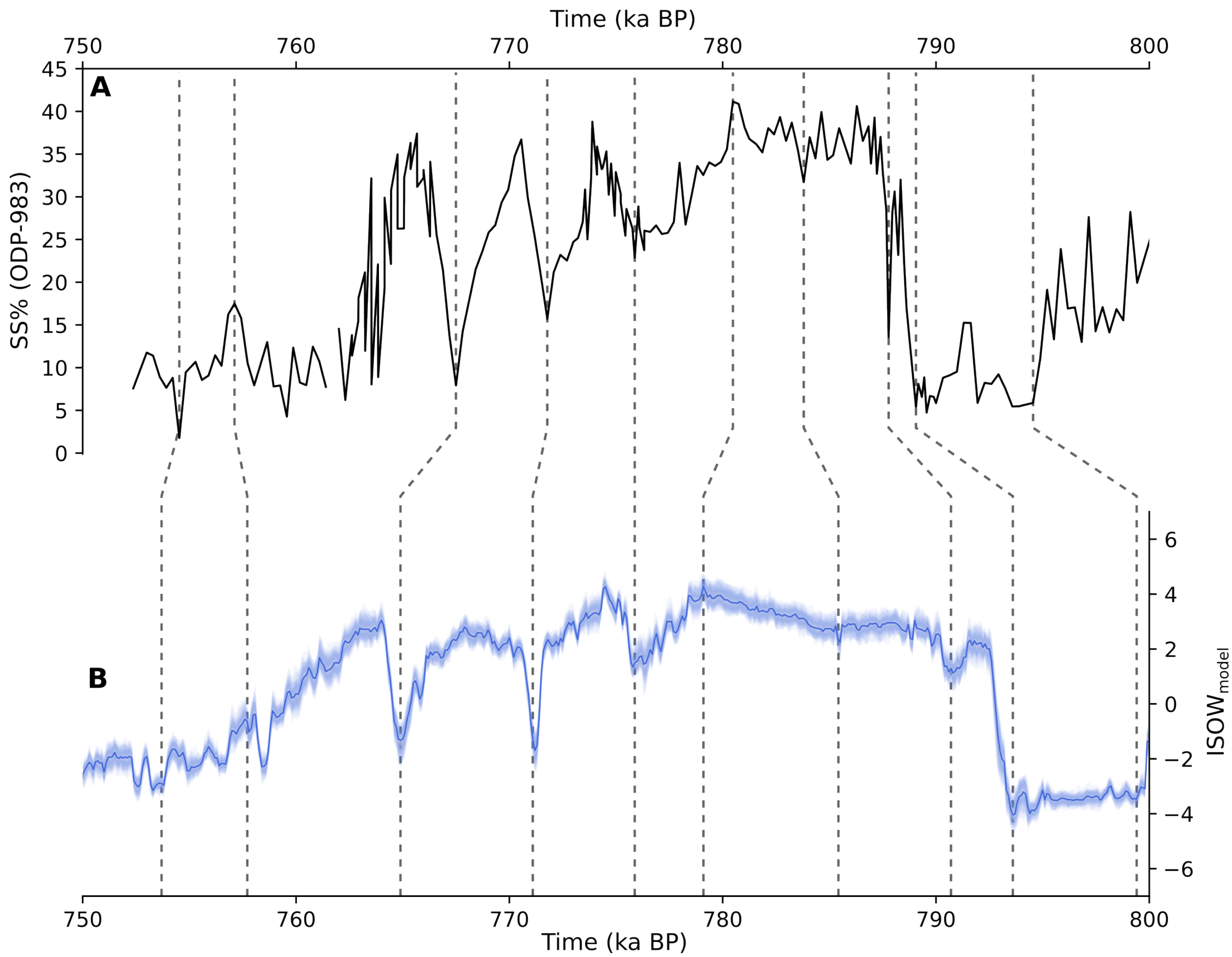

Fig. S11.

Comparison between the ISOW$_{model}$ stack and the SS% from ODP site 983 (*86*). The comparison is shown for the 750 to 800 ka time interval. Grey vertical lines indicate millennial-scale correspondence between the two records. Shaded blue envelope represents the ISOW$_{model}$ uncertainty (from percentiles 1 to 99). Records are plotted in their respective chronologies.

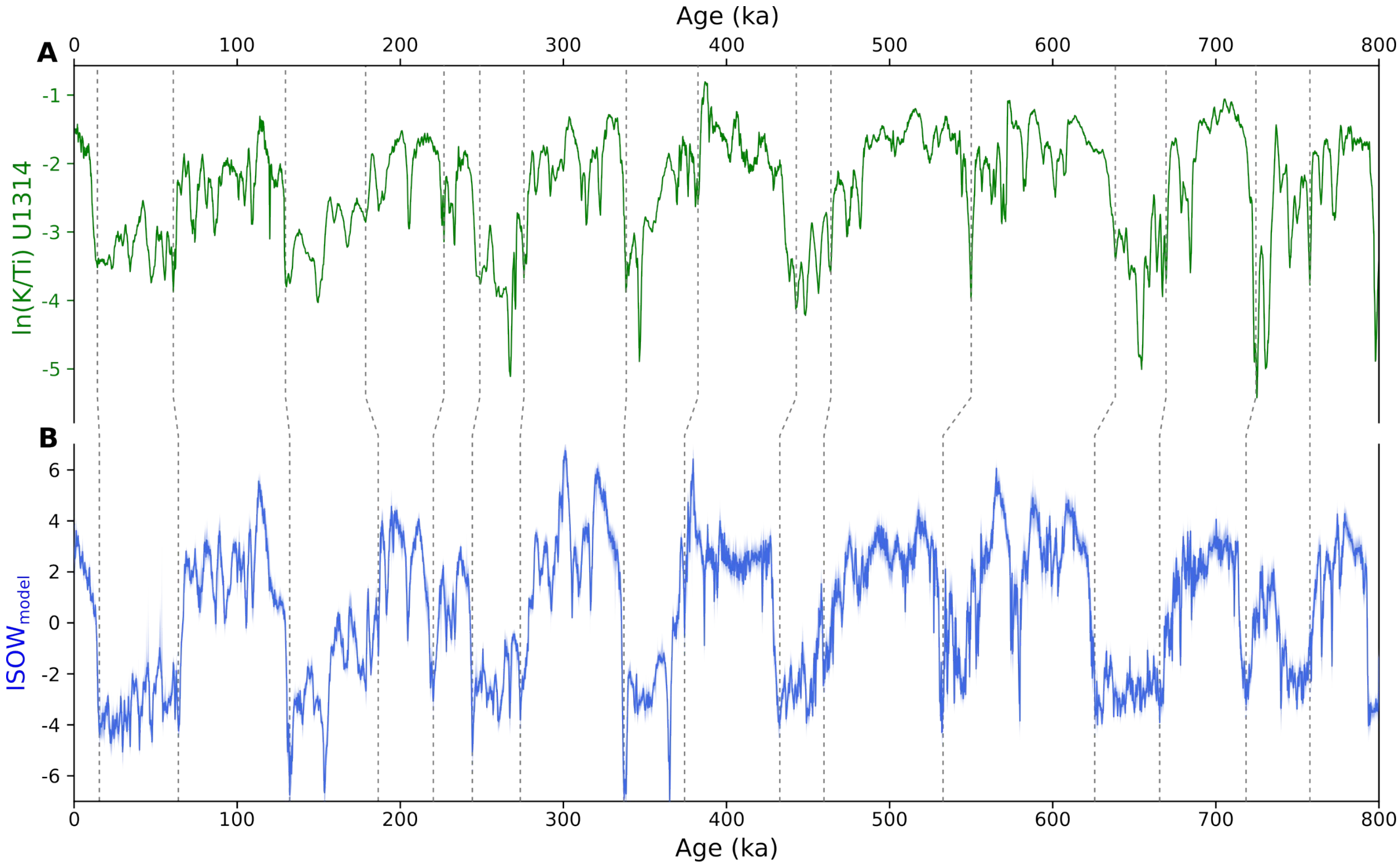

Fig. S12.

Comparison between ISOW$_{model}$ and ln(K/Ti) from IODP site U1314 (*39*) over the last 800 ka. Grey vertical lines indicate the correspondence between the two records. Shaded blue envelope represents the ISOW$_{model}$ uncertainty (from percentiles 1 to 99). Blue numbers indicate glacial MIS, red numbers indicate interglacial MIS. Records are plotted in their respective chronologies.

Table S1.

Metrics of the 5-fold cross-validation.

	**RMSE**	**R^2**	**r (Pearson)**	**r (Spearman)**
**Fold 1**	0.548	0.966	0.983	0.982
**Fold 2**	0.535	0.966	0.983	0.983
**Fold 3**	0.651	0.949	0.975	0.973
**Fold 4**	0.531	0.967	0.984	0.983
**Fold 5**	0.492	0.971	0.986	0.985
**Mean**	***0.551***	***0.964***	***0.982***	***0.981***

Table S2.

Mean metrics of the 5-fold cross-validation with different block-based splits between training and validation datasets.

	Blocks (ka)	MSE	R^2	*r* (Pearson)	*r* (Spearman)
**Random**	0	0.551	0.964	0.982	0.981
**0.5-ka blocks**	0.5	1.036	0.871	0.934	0.930
**1-ka blocks**	1	1.328	0.783	0.889	0.885
**2-ka blocks**	2	1.544	0.715	0.847	0.834
**3-ka blocks**	3	1.726	0.631	0.804	0.783
**4-ka blocks**	4	1.789	0.603	0.785	0.767
**5-ka blocks**	5	1.863	0.563	0.776	0.747
**7.5-ka blocks**	7.5	1.972	0.499	0.720	0.648
**10-ka blocks**	10	1.937	0.465	0.725	0.703
**20-ka blocks**	20	2.087	0.426	0.704	0.679
**50-ka blocks**	50	2.119	0.307	0.681	0.641

Table S3.

Detected thresholds with three different smoothing methods.

Smoothing method	Weak-to-strong		Strong-to-weak	
	CO_2 (ppm)	*Sea-level (m)*	*CO_2 (ppm)*	*Sea-level (m)*
Spline	238 to 266	-58 to -37	205 to 214	-64 to -51
Low-band (1/12 ka^{-1})	235 to 258	-63 to -45	202 to 211	-65 to -52
Moving average (7 ka)	242 to 265	-55 to -39	201 to 208	-57 to -50
Mean	***251***	***-50***	***206***	***-57***

Data S1. (separate file)

Data S1 is the 1st to 99th percentiles of the $ISOW_{hybrid}$ stack. It is based on $ISOW_{data}$ and $ISOW_{model}$ stacks for the 0 to 380 ka and 380 to 800 ka periods, respectively.

Data S2. (separate file)

Data S2 is the $ISOW_{hybrid}$ stack (median) with the $\pm\sigma$ and $\pm 2\sigma$. It is based on $ISOW_{data}$ and $ISOW_{model}$ stacks for the 0 to 380 ka and 380 to 800 ka periods, respectively.

REFERENCES

1. M. W. Buckley, J. Marshall, Observations, inferences, and mechanisms of the Atlantic Meridional Overturning Circulation: A review. *Rev. Geophys.* **54**, 5–63 (2016).

2. W. E. Johns, S. Elipot, D. A. Smeed, B. Moat, B. King, D. L. Volkov, R. H. Smith, Towards two decades of Atlantic Ocean mass and heat transports at 26.5° N. *Philos. Trans. A Math. Phys. Eng. Sci.* **381**, 20220188 (2023).

3. S. Zou, A. Bower, H. Furey, M. Susan Lozier, X. Xu, Redrawing the Iceland–Scotland Overflow Water pathways in the North Atlantic. *Nat. Commun.* **11**, 1890 (2020).

4. T. Eldevik, J. E. Ø. Nilsen, D. Iovino, K. A. Olsson, A. B. Sandø, H. Drange, Observed sources and variability of Nordic seas overflow. *Nat. Geosci.* **2**, 406–410 (2009).

5. N. Daniault, H. Mercier, P. Lherminier, A. Sarafanov, A. Falina, P. Zunino, F. F. Pérez, A. F. Ríos, B. Ferron, T. Huck, V. Thierry, S. Gladyshev, The northern North Atlantic Ocean mean circulation in the early 21st century. *Prog. Oceanogr.* **146**, 142–158 (2016).

6. M. S. Lozier, A. S. Bower, H. H. Furey, K. L. Drouin, X. Xu, S. Zou, Overflow water pathways in the North Atlantic. *Prog. Oceanogr.* **208**, 102874 (2022).

7. M. S. Lozier, Overturning in the subpolar North Atlantic: A review. *Philos. Trans. A Math. Phys. Eng. Sci.* **381**, 20220191 (2023).

8. S. Zou, S. Lozier, W. Zenk, A. Bower, W. Johns, Observed and modeled pathways of the Iceland Scotland Overflow Water in the eastern North Atlantic. *Prog. Oceanogr.* **159**, 211–222 (2017).

9. W. E. Johns, M. Devana, A. Houk, S. Zou, Moored observations of the Iceland-Scotland overflow plume along the eastern flank of the Reykjanes Ridge. *J. Geophys. Res. Oceans* **126**, e2021JC017524 (2021).

10. M. Devana, W. Johns, Structure and variability of Iceland Scotland Overflow Water transport in the western Iceland Basin. *J. Geophys. Res. Oceans* **129**, e2023JC020107 (2024).

11. N. Stevenard, C. Kissel, A. Govin, C. Wandres, Deep North Atlantic circulation strength: Glacial-interglacial variability over the last 400,000 years. *Quat. Sci. Rev.* **345**, 109011 (2024).

12. P. Blaser, C. Waelbroeck, D. J. R. Thornalley, J. Lippold, F. Pöppelmeier, S. Kaboth-Bahr, J. Repschläger, S. L. Jaccard, Prevalent North Atlantic Deep Water during the Last Glacial Maximum and Heinrich Stadial 1. *Nat. Geosci.* **18**, 410–416 (2025).

13. C. Kissel, C. Laj, L. Labeyrie, T. Dokken, A. Voelker, D. Blamart, Rapid climatic variations during marine isotopic stage 3: Magnetic analysis of sediments from Nordic Seas and North Atlantic. *Earth Planet. Sci. Lett.* **171**, 489–502 (1999).

14. H. Stommel, Thermohaline convection with two stable regimes of flow. *Tellus* **13**, 224–230 (1961).

15. S. Rahmstorf, On the freshwater forcing and transport of the Atlantic thermohaline circulation. *Clim. Dyn.* **12**, 799–811 (1996).

16. A. Ganopolski, S. Rahmstorf, Rapid changes of glacial climate simulated in a coupled climate model. *Nature* **409**, 153–158 (2001).

17. M. Willeit, A. Ganopolski, Generalized stability landscape of the Atlantic meridional overturning circulation. *Earth Syst. Dyn.* **15**, 1417–1434 (2024).

18. W. Weijer, W. Cheng, S. S. Drijfhout, A. V. Fedorov, A. Hu, L. C. Jackson, W. Liu, E. L. McDonagh, J. V. Mecking, J. Zhang, Stability of the Atlantic Meridional Overturning Circulation: A review and synthesis. *J. Geophys. Res. Oceans* **124**, 5336–5375 (2019).

19. Y. M. Romé, R. F. Ivanovic, L. J. Gregoire, D. Swingedouw, S. Sherriff-Tadano, R. Börner, Simulated millennial-scale climate variability driven by a convection–advection oscillator. *Clim. Dyn.* **63**, 150 (2025).

20. X. Zhang, G. Lohmann, G. Knorr, C. Purcell, Abrupt glacial climate shifts controlled by ice sheet changes. *Nature* **512**, 290–294 (2014).

21. X. Zhang, G. Knorr, G. Lohmann, S. Barker, Abrupt North Atlantic circulation changes in response to gradual CO_2 forcing in a glacial climate state. *Nat. Geosci.* **10**, 518–523 (2017).

22. X. Zhang, S. Barker, G. Knorr, G. Lohmann, R. Drysdale, Y. Sun, D. Hodell, F. Chen, Direct astronomical influence on abrupt climate variability. *Nat. Geosci.* **14**, 819–826 (2021).

23. X. Zhang, M. Prange, Stability of the Atlantic overturning circulation under intermediate (MIS3) and full glacial (LGM) conditions and its relationship with Dansgaard-Oeschger climate variability. *Quat. Sci. Rev.* **242**, 106443 (2020).

24. M. Klockmann, U. Mikolajewicz, J. Marotzke, Two AMOC states in response to decreasing greenhouse gas concentrations in the coupled climate model MPI-ESM. *J. Clim.* **31**, 7969–7984 (2018).

25. G. Vettoretti, P. Ditlevsen, M. Jochum, S. O. Rasmussen, Atmospheric CO_2 control of spontaneous millennial-scale ice age climate oscillations. *Nat. Geosci.* **15**, 300–306 (2022).

26. I. Malmierca-Vallet, L. C. Sime, P. J. Valdes, M. Klockmann, G. Vettoretti, J. Slattery, The impact of CO_2 and climate state on whether Dansgaard–Oeschger type oscillations occur in climate models. *Geophys. Res. Lett.* **51**, e2024GL110068 (2024).

27. S. Barker, G. Knorr, Millennial scale feedbacks determine the shape and rapidity of glacial termination. *Nat. Commun.* **12**, 2273 (2021).

28. S. Barker, G. Knorr, A systematic role for extreme ocean-atmosphere oscillations in the development of glacial conditions since the Mid Pleistocene Transition. *Paleoceanogr. Paleoclimatol.* **38**, e2023PA004690 (2023).

29. Y. Sun, J. F. McManus, S. C. Clemens, X. Zhang, H. Vogel, D. A. Hodell, F. Guo, T. Wang, X. Liu, Z. An, Persistent orbital influence on millennial climate variability through the Pleistocene. *Nat. Geosci.* **14**, 812–818 (2021).

30. J.-M. Gherardi, L. Labeyrie, S. Nave, R. Francois, J. F. McManus, E. Cortijo, Glacial-interglacial circulation changes inferred from $^{231}Pa/^{230}Th$ sedimentary record in the North Atlantic region. *Paleoceanography* **24**, 2008PA001696 (2009).

31. E. Böhm, J. Lippold, M. Gutjahr, M. Frank, P. Blaser, B. Antz, J. Fohlmeister, N. Frank, M. B. Andersen, M. Deininger, Strong and deep Atlantic meridional overturning circulation during the last glacial cycle. *Nature* **517**, 73–76 (2015).

32. L. G. Henry, J. F. McManus, W. B. Curry, N. L. Roberts, A. M. Piotrowski, L. D. Keigwin, North Atlantic ocean circulation and abrupt climate change during the last glaciation. *Science* **353**, 470–474 (2016).

33. H. C. Ng, L. F. Robinson, J. F. McManus, K. J. Mohamed, A. W. Jacobel, R. F. Ivanovic, L. J. Gregoire, T. Chen, Coherent deglacial changes in western Atlantic Ocean circulation. *Nat. Commun.* **9**, 2947 (2018).

34. F. Süfke, F. Pöppelmeier, T. J. Goepfert, M. Regelous, A. Koutsodendris, P. Blaser, M. Gutjahr, J. Lippold, Constraints on the Northwestern Atlantic Deep Water circulation from $^{231}Pa/^{230}Th$ during the last 30,000 years. *Paleoceanogr. Paleoclimatol.* **34**, 1945–1958 (2019).

35. F. Pöppelmeier, A. Jeltsch-Thömmes, J. Lippold, F. Joos, T. F. Stocker, Multi-proxy constraints on Atlantic circulation dynamics since the last ice age. *Nat. Geosci.* **16**, 349–356 (2023).

36. C. Kissel, C. Laj, A. M. Piotrowski, S. L. Goldstein, S. R. Hemming, Millennial-scale propagation of Atlantic deep waters to the glacial Southern Ocean. *Paleoceanography* **23**, 2008PA001624 (2008).

37. C. Kissel, A. Van Toer, C. Laj, E. Cortijo, E. Michel, Variations in the strength of the North Atlantic bottom water during Holocene. *Earth Planet. Sci. Lett.* **369-370**, 248–259 (2013).

38. D. J. R. Thornalley, M. Blaschek, F. J. Davies, S. Praetorius, D. W. Oppo, J. F. McManus, I. R. Hall, H. Kleiven, H. Renssen, I. N. McCave, Long-term variations in Iceland–Scotland overflow strength during the Holocene. *Clim. Past* **9**, 2073–2084 (2013).

39. J. Grützner, S. M. Higgins, Threshold behavior of millennial scale variability in deep water hydrography inferred from a 1.1 Ma long record of sediment provenance at the southern Gardar Drift. *Paleoceanography* **25**, 17 (2010).

40. C. J. Berends, B. De Boer, R. S. W. Van De Wal, Reconstructing the evolution of ice sheets, sea level, and atmospheric CO_2 during the past 3.6 million years. *Clim. Past* **17**, 361–377 (2021).

41. B. Bereiter, S. Eggleston, J. Schmitt, C. Nehrbass-Ahles, T. F. Stocker, H. Fischer, S. Kipfstuhl, J. Chappellaz, Revision of the EPICA Dome C CO_2 record from 800 to 600 kyr before present. *Geophys. Res. Lett.* **42**, 542–549 (2015).

42. E. Legrain, E. Capron, L. Menviel, A. Wohleber, F. Parrenin, G. Teste, A. Landais, M. Bouchet, R. Grilli, C. Nehrbass-Ahles, L. Silva, H. Fischer, T. F. Stocker, Centennial-scale variations in the carbon cycle enhanced by high obliquity. *Nat. Geosci.* **17**, 1154–1161 (2024).

43. S. Barker, G. Knorr, R. L. Edwards, F. Parrenin, A. E. Putnam, L. C. Skinner, E. Wolff, M. Ziegler, 800,000 years of abrupt climate variability. *Science* **334**, 347–351 (2011).

44. S. Barker, X. Zhang, L. Jonkers, S. Lordsmith, S. Conn, G. Knorr, Strengthening Atlantic inflow across the Mid-Pleistocene Transition. *Paleoceanogr. Paleoclimatol.* **36**, e2020PA004200 (2021).

45. D. A. Hodell, S. J. Crowhurst, L. Lourens, V. Margari, J. Nicolson, J. E. Rolfe, L. C. Skinner, N. C. Thomas, P. C. Tzedakis, M. J. Mleneck-Vautravers, E. W. Wolff, A 1.5-million-year record of orbital and millennial climate variability in the North Atlantic. *Clim. Past* **19**, 607–636 (2023).

46. A. Berger, M. F. Loutre, Insolation values for the climate of the last 10 million years. *Quat. Sci. Rev.* **10**, 297–317 (1991).

47. S. M. Lundberg, S.-I. Lee, A unified approach to interpreting model predictions. *Adv. Neural Inf. Proces. Syst.* **30**, 4768–4777 (2017).

48. W. B. Curry, D. W. Oppo, Glacial water mass geometry and the distribution of $\delta^{13}C$ of ΣCO_2 in the western Atlantic Ocean. *Paleoceanography* **20**, 2004PA001021 (2005).

49. J. N. W. Howe, A. M. Piotrowski, T. L. Noble, S. Mulitza, C. M. Chiessi, G. Bayon, North Atlantic Deep Water production during the Last Glacial Maximum. *Nat. Commun.* **7**, 11765 (2016).

50. J. Lynch-Stieglitz, The Atlantic Meridional Overturning Circulation and abrupt climate change. *Ann. Rev. Mar. Sci.* **9**, 83–104 (2017).

51. S. Gu, Z. Liu, H. C. Ng, J. Lynch-Stieglitz, J. F. McManus, M. Spall, A. Jahn, C. He, L. Li, M. Yan, L. Wu, Open ocean convection drives enhanced eastern pathway of the glacial Atlantic Meridional Overturning Circulation. *Proc. Natl. Acad. Sci. U. S. A.* **121**, e2405051121 (2024).

52. L. C. Menviel, P. Spence, L. C. Skinner, K. Tachikawa, T. Friedrich, L. Missiaen, J. Yu, Enhanced mid-depth southward transport in the Northeast Atlantic at the Last Glacial Maximum despite a weaker AMOC. *Paleoceanogr. Paleoclimatol.* **35**, e2019PA003793 (2020).

53. A. Paul, S. Mulitza, R. Stein, M. Werner, A global climatology of the ocean surface during the Last Glacial Maximum mapped on a regular grid (GLOMAP). *Clim. Past* **17**, 805–824 (2021).

54. L. Gerber, J. Lippold, J. Repschläger, O. Friedrich, P. Testorf, M. Ehnis, F. Süfke, P. Blaser, F. Pöppelmeier, S. L. Jaccard, Water-depth-dependent $^{231}Pa/^{230}Th$ responses in the central North Atlantic since the Last Glacial Maximum. *Paleoceanogr. Paleoclimatol.* **41**, e2026PA005494 (2026).

55. A. Guihou, S. Pichat, S. Nave, A. Govin, L. Labeyrie, E. Michel, C. Waelbroeck, Late slowdown of the Atlantic Meridional Overturning Circulation during the Last Glacial Inception: New constraints from sedimentary ($^{231}Pa/^{230}Th$). *Earth Planet. Sci. Lett.* **289**, 520–529 (2010).

56. A. Guihou, S. Pichat, A. Govin, S. Nave, E. Michel, J.-C. Duplessy, P. Telouk, L. Labeyrie, Enhanced Atlantic Meridional Overturning Circulation supports the Last Glacial Inception. *Quat. Sci. Rev.* **30**, 1576–1582 (2011).

57. E. V. Galaasen, U. S. Ninnemann, A. Kessler, N. Irvalı, Y. Rosenthal, J. Tjiputra, N. Bouttes, D. M. Roche, H. K. F. Kleiven, D. A. Hodell, Interglacial instability of North Atlantic Deep Water ventilation. *Science* **367**, 1485–1489 (2020).

58. J. F. McManus, D. W. Oppo, J. L. Cullen, A 0.5-million-year record of millennial-scale climate variability in the North Atlantic. *Science* **283**, 971–975 (1999).

59. F. Sévellec, A. V. Fedorov, Unstable AMOC during glacial intervals and millennial variability: The role of mean sea ice extent. *Earth Planet. Sci. Lett.* **429**, 60–68 (2015).

60. M. Wary, F. Eynaud, D. Swingedouw, V. Masson-Delmotte, J. Matthiessen, C. Kissel, J. Zumaque, L. Rossignol, J. Jouzel, Regional seesaw between the North Atlantic and Nordic Seas during the last glacial abrupt climate events. *Clim. Past* **13**, 729–739 (2017).

61. R. M. Van Westen, H. A. Dijkstra, Asymmetry of AMOC hysteresis in a state-of-the-art global climate model. *Geophys. Res. Lett.* **50**, e2023GL106088 (2023).

62. G. H. Denton, R. F. Anderson, J. R. Toggweiler, R. L. Edwards, J. M. Schaefer, A. E. Putnam, The last glacial termination. *Science* **328**, 1652–1656 (2010).

63. U. Hoff, T. L. Rasmussen, R. Stein, M. M. Ezat, K. Fahl, Sea ice and millennial-scale climate variability in the Nordic seas 90 kyr ago to present. *Nat. Commun.* **7**, 1508–1530 (2016).

64. S. Nuber, J. W. B. Rae, X. Zhang, M. B. Andersen, M. D. Dumont, H. T. Mithan, Y. Sun, B. De Boer, I. R. Hall, S. Barker, Indian Ocean salinity build-up primes deglacial ocean circulation recovery. *Nature* **617**, 306–311 (2023).

65. Q. Z. Yin, A. Berger, Individual contribution of insolation and CO_2 to the interglacial climates of the past 800,000 years. *Clim. Dyn.* **38**, 709–724 (2012).

66. Q. Yin, Insolation-induced mid-Brunhes transition in Southern Ocean ventilation and deep-ocean temperature. *Nature* **494**, 222–225 (2013).

67. L. E. Lisiecki, M. E. Raymo, A Pliocene-Pleistocene stack of 57 globally distributed benthic $\delta^{18}O$ records. *Paleoceanography* **20**, 2004PA001071 (2005).

68. R. M. Spratt, L. E. Lisiecki, A Late Pleistocene sea level stack. *Clim. Past* **12**, 1079–1092 (2016).

69. T. M. Cronin, G. S. Dwyer, E. K. Caverly, J. Farmer, L. H. DeNinno, J. Rodriguez-Lazaro, L. Gemery, Enhanced Arctic amplification began at the Mid-Brunhes Event ~400,000 years ago. *Sci. Rep.* **7**, 14475 (2017).

70. T. Caley, J. Giraudeau, B. Malaizé, L. Rossignol, C. Pierre, Agulhas leakage as a key process in the modes of Quaternary climate changes. *Proc. Natl. Acad. Sci. U. S. A.* **109**, 6835–6839 (2012).

71. M. Årthun, A. Brakstad, J. Dörr, H. L. Johnson, C. Mans, S. Semper, K. Våge, Atlantification drives recent strengthening of the Arctic overturning circulation. *Sci. Adv.* **11**, eadu1794 (2025).

72. M. Cini, G. Zappa, F. Ragone, S. Corti, Noise-shaped hysteresis cycles of the AMOC under increasing CO_2 forcing. *Chaos* **35**, 023167 (2025).

73. D. Veres, L. Bazin, A. Landais, H. T. M. Kele, B. Lemieux-Dudon, F. Parrenin, P. Martinerie, E. Blayo, T. Blunier, E. Capron, J. Chappellaz, S. O. Rasmussen, M. Severi, A. Svensson, B. Vinther, E. W. Wolff, The Antarctic ice core chronology (AICC2012): An optimized multi-parameter and multi-site dating approach for the last 120 thousand years. *Clim. Past* **9**, 1733–1748 (2013).

74. L. Bazin, A. Landais, B. Lemieux-Dudon, H. T. M. Kele, D. Veres, F. Parrenin, P. Martinerie, C. Ritz, E. Capron, V. Lipenkov, M.-F. Loutre, D. Raynaud, B. Vinther, A. Svensson, S. O. Rasmussen, M. Severi, T. Blunier, M. Leuenberger, H. Fischer, V. Masson-Delmotte, J. Chappellaz, E. Wolff, An optimized multi-proxy, multi-site Antarctic ice and gas orbital chronology (AICC2012): 120–800 ka. *Clim. Past* **9**, 1715–1731 (2013).

75. S. Barker, J. Chen, X. Gong, L. Jonkers, G. Knorr, D. Thornalley, Icebergs not the trigger for North Atlantic cold events. *Nature* **520**, 333–336 (2015).

76. C. Nehrbass-Ahles, J. Shin, J. Schmitt, B. Bereiter, F. Joos, A. Schilt, L. Schmidely, L. Silva, G. Teste, R. Grilli, J. Chappellaz, D. Hodell, H. Fischer, T. F. Stocker, Abrupt CO_2 release to

the atmosphere under glacial and early interglacial climate conditions. *Science* **369**, 1000–1005 (2020).

77. Q. Z. Yin, Z. P. Wu, A. Berger, H. Goosse, D. Hodell, Insolation triggered abrupt weakening of Atlantic circulation at the end of interglacials. *Science* **373**, 1035–1040 (2021).

78. D. Hodell, L. Lourens, S. Crowhurst, T. Konijnendijk, R. Tjallingii, F. Jiménez-Espejo, L. Skinner, P. C. Tzedakis, F. Abrantes, G. D. Acton, C. A. Alvarez Zarikian, A. Bahr, B. Balestra, E. L. Barranco, G. Carrara, E. Ducassou, R. D. Flood, J.-A. Flores, S. Furota, J. Grimalt, P. Grunert, J. Hernández-Molina, J. K. Kim, L. A. Krissek, J. Kuroda, B. Li, J. Lofi, V. Margari, B. Martrat, M. D. Miller, F. Nanayama, N. Nishida, C. Richter, T. Rodrigues, F. J. Rodríguez-Tovar, A. C. F. Roque, M. F. Sanchez Goñi, F. J. Sierro Sánchez, A. D. Singh, C. R. Sloss, D. A. V. Stow, Y. Takashimizu, A. Tzanova, A. Voelker, C. Xuan, T. Williams, A reference time scale for Site U1385 (Shackleton Site) on the SW Iberian Margin. *Glob. Planet. Change* **133**, 49–64 (2015).

79. F. Pedregosa, G. Varoquaux, A. Gramfort, V. Michel, B. Thirion, O. Grisel, M. Blondel, P. Prettenhofer, R. Weiss, V. Dubourg, J. Vanderplas, A. Passos, D. Cournapeau, M. Brucher, M. Perrot, E. Duchesnay, Scikit-learn: Machine learning in Python. *J. Mach. Learn. Res.* **12**, 2825–2830 (2011).

80. T. Chen, C. Guestrin, "XGBoost: A scalable tree boosting system," in *Proceedings of the 22nd ACM SIGKDD International Conference on Knowledge Discovery and Data Mining* (Association for Computing Machinery, 2016), pp. 785–794.

81. T. Akiba, S. Sano, T. Yanase, T. Ohta, M. Koyama, "Optuna: A next-generation hyperparameter optimization framework," in *Proceedings of the 25th ACM SIGKDD International Conference on Knowledge Discovery & Data Mining* (Association for Computing Machinery, 2019), pp. 2623–2631.

82. P. Virtanen, R. Gommers, T. E. Oliphant, M. Haberland, T. Reddy, D. Cournapeau, E. Burovski, P. Peterson, W. Weckesser, J. Bright, S. J. Van Der Walt, M. Brett, J. Wilson, K. J. Millman, N. Mayorov, A. R. J. Nelson, E. Jones, R. Kern, E. Larson, C. J. Carey, İ. Polat, Y. Feng, E. W. Moore, J. VanderPlas, D. Laxalde, J. Perktold, R. Cimrman, I. Henriksen, E. A.

Quintero, C. R. Harris, A. M. Archibald, A. H. Ribeiro, F. Pedregosa, P. Van Mulbregt, SciPy 1.0 Contributors, SciPy 1.0: Fundamental algorithms for scientific computing in Python. *Nat. Methods* **17**, 261–272 (2020).

83. D. Khider, J. Emile-Geay, F. Zhu, A. James, J. Landers, V. Ratnakar, Y. Gil, Pyleoclim: Paleoclimate timeseries analysis and visualization with Python. *Paleoceanogr. Paleoclimatol.* **37**, e2022PA004509 (2022).

84. W. Dansgaard, S. J. Johnsen, H. B. Clausen, D. Dahl-Jensen, N. S. Gundestrup, C. U. Hammer, C. S. Hvidberg, J. P. Steffensen, A. E. Sveinbjörnsdottir, J. Jouzel, G. Bond, Evidence for general instability of past climate from a 250-kyr ice-core record. *Nature* **364**, 218–220 (1993).

85. D. J. R. Thornalley, S. Barker, J. Becker, I. R. Hall, G. Knorr, Abrupt changes in deep Atlantic circulation during the transition to full glacial conditions. *Paleoceanography* **28**, 253–262 (2013).

86. H. F. Kleiven, I. R. Hall, I. N. McCave, G. Knorr, E. Jansen, Coupled deep-water flow and climate variability in the middle Pleistocene North Atlantic. *Geology* **39**, 343–346 (2011).

87. N. Stevenard, A. Govin, C. Kissel, A. Van Toer, Correction of the IRD influence for paleo-current flow speed reconstructions in hemipelagic sediments. *Paleoceanogr. Paleoclimatol.* **38**, e2022PA004500 (2023).
